# The kinetic mechanisms of fast-decay red-fluorescent genetically encoded calcium indicators

**DOI:** 10.1074/jbc.RA118.004543

**Published:** 2019-01-16

**Authors:** Silke Kerruth, Catherine Coates, Céline D. Dürst, Thomas G. Oertner, Katalin Török

**Affiliations:** From the ‡ Molecular and Clinical Sciences Research Institute, St. George's, University of London, London SW17 0RE, United Kingdom and; the § Institute for Synaptic Physiology, Center for Molecular Neurobiology Hamburg, 20251 Hamburg, Germany

**Keywords:** calcium, calcium imaging, fluorescence, kinetics, biosensor

## Abstract

Genetically encoded calcium indicators (GECIs) are useful reporters of cell-signaling, neuronal, and network activities. We have generated novel fast variants and investigated the kinetic mechanisms of two recently developed red-fluorescent GECIs (RGECIs), mApple-based jRGECO1a and mRuby-based jRCaMP1a. In the formation of fluorescent jRGECO1a and jRCaMP1a complexes, calcium binding is followed by rate-limiting isomerization. However, fluorescence decay of calcium-bound jRGECO1a follows a different pathway from its formation: dissociation of calcium occurs first, followed by the peptide, similarly to GCaMP-s. In contrast, fluorescence decay of calcium-bound jRCaMP1a occurs by the reversal of the on-pathway: peptide dissociation is followed by calcium. The mechanistic differences explain the generally slower off-kinetics of jRCaMP1a-type indicators compared with GCaMP-s and jRGECO1a-type GECI: the fluorescence decay rate of f-RCaMP1 was 21 s^−1^, compared with 109 s^−1^ for f-RGECO1 and f-RGECO2 (37 °C). Thus, the CaM–peptide interface is an important determinant of the kinetic responses of GECIs; however, the topology of the structural link to the fluorescent protein demonstrably affects the internal dynamics of the CaM–peptide complex. In the dendrites of hippocampal CA3 neurons, f-RGECO1 indicates calcium elevation in response to a 100 action potential train in a linear fashion, making the probe particularly useful for monitoring large-amplitude, fast signals, *e.g.* those in dendrites, muscle cells, and immune cells.

## Introduction

Monitoring Ca^2+^ signaling with fluorescent indicators has been widely used to readout neuronal activity (for an overview see Refs. 1–3). Genetically encoded calcium indicators (GECIs)^2^ are noninvasive and targetable to cell types and cellular compartments. Initially, GECI based on FRET (*e.g.* Chameleon) (4) were followed by single-fluorophore sensors based on circularly permuted (cp) fluorescent proteins (FPs) (GCaMP, RCaMP, and Flash-pericam), which were better suited for two-photon imaging (5, 6). Further mutation of the cpFP or replacement with other red fluorescent proteins led to the generation of variants with different excitation and emission spectra (B-CaMP, B-GECO, YCaMP, CyCaMP, etc.) (7, 8). Those probes are potentially useful for multicolor imaging, as well as optogenetic experiments, because their emission wavelength does not overlap with the blue light used to excite light-gated channels or pumps that are simultaneously expressed (9–11). Since the publication of the first GCaMP (5), intensive research has been conducted toward improving the properties of GECIs (7, 8, 12–16).

**Figure 1.**
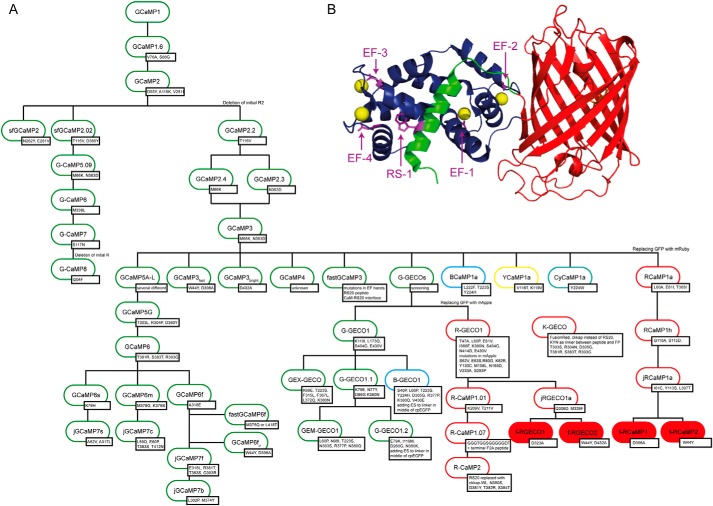
*A*, GECI family tree. Shown are the most famous variants of published data. The inserted mutations are shown in the *small boxes* next to the names. The numbering is according to GCaMP1 (also for RGECI) for better comparison of the mutations done in the RS20 (positions 41–59) and CaM (positions 305–451) domain. Starting from GCaMP1 (5), mutations in the cpEGFP were introduced to make the construct more stable at 37 °C and also preventing dimerization, leading to the development of GCaMP1.6 and GCaMP2 (38). From this construct the deletion of R2 and a few other mutations led to the improved sensor GCaMP3 (12). GCaMP3 is the base of many different GCaMPs as well as the ones with shifted emission spectra, like BCaMP1a, YCaMP1a, and CyCaMP1a (7) or the family of G-GECO, which was used to derive sensors named B-GECO and R-GECO (16). In addition to expanding the color palette, probes with higher fluorescence intensity and improved kinetics were generated, such as the GCaMP5 (14), GCaMP6 (13), and the recently published jGCaMP7 family (39). Others focused on generating sensors with very fast kinetics like fastGCaMP3 (21) and GCaMP3_fast_ (23), as well as fastGCaMP6f (22) and GCaMP6f_u_ (24). Parallel to the development of the GCaMP3 branch, a second branch evolved on the basis of GCaMP2 that also included the mutations of GCaMP3 and the mutation of the superfast GFP, generating GCaMP5.09, GCaMP6, and the very bright GCaMP7, as well as GCaMP8 that also, like all GCaMP3-based sensors, misses R2 (15). For the development of the red probe–based RCaMP1a and R-GECO1 cpEGFP was replaced by mRuby or mApple, respectively. Mutations in the RFPs are explicated. On their basis the variants jRGECO1a and jRCaMP1a were generated (8); they are the parental sensors for the probes developed in this work (highlighted in *red*). Confusingly, sensors also named R-CaMP, based on RGECO1, were generated that also carry mApple as their fluorescent protein but differ by a few mutations (R-CaMP1.01) or contain a C-terminal FA peptide (R-CaMP1.07) (17). Based on this probe, R-CaMP2 was published in which the RS20 peptide was replaced by a chimera of the CaM-binding sequence of CaM-dependent kinase kinases α and β (9). Recently a novel red fluorescent GECI, named K-GECO, was developed that is based on a fluorescent protein from *Discosoma* sp. mushroom and the rat CaM-dependent kinase kinase peptide (ckkap) (40). *B*, crystal structure of jRCaMP1a (PDB code 3U0K (7)). CaM is shown in *dark blue* with bound Ca^2+^ as *yellow spheres*. RS20 is shown in *green* and circularly permuted mRuby is colored *red*. The mutation sites described here are shown as *sticks* and are highlighted in *purple*.

Red GECIs (RGECIs) follow the design of GCaMPs because they are composed of a cp red fluorescent protein (cpRFP) that is fused to the smooth muscle myosin light-chain kinase peptide RS20 at its N terminus and to calmodulin (CaM) at its C terminus. The RGECO variants are based on the cp mApple, whereas the RCaMPs contain cp mRuby, with the exception of R-CaMPs, which are also based on cp mApple (9, 17). Probes that are based on mApple usually show higher dynamic range; however, excitation by blue light leads to photoswitching, generating a fluorescence output that can lead to artifacts (7, 8). Thus, although RGECOs have higher fluorescence signals, RCaMPs are useful for optogenetic experiments (18, 19). We have drawn up a “family tree” of GECIs and derived variants to provide an overview of the mutation sites and lineage of probe development (Fig. 1).

Because of the core cpFP, without Ca^2+^, the intrinsic chromophore exists mostly in its protonated neutral state that shows no fluorescence. Upon Ca^2+^ binding to CaM, a hydrophobic interface is exposed that binds to the RS20 peptide forcing changes in the conformation of the β-barrel structure of the cpRFP, leading to deprotonation of the chromophore and formation of the anionic state that has high fluorescence emission. Previous studies on GCaMP3 and GCaMP6f showed slow fluorescence rise and decay in response to Ca^2+^ concentration changes, to a large extent caused by the steric constraints imposed by cpFP interjecting between CaM and the RS20 peptide as well as their strong binding with a *K_d_* in the nanomolar range (20). Targeted mutations in the RS20 and CaM domains has led to fastGCaMP3 (21) and fastGCaMP6f (22), as well as GCaMP3_fast_ (23) and GCaMP6f_u_ (24). GCaMP6f_u_ is the fastest GCaMP so far, with a limiting *k*_on_ of 142 s^−1^ and a *k*_off_ of 89 s^−1^ at 20 °C (24). These new GECIs can be used for more faithful temporal monitoring of Ca^2+^ transients associated with synaptic transmission and activation of skeletal and cardiac muscles, which occur on the millisecond time scale.

Helassa *et al.* (24) and Sun *et al.* (21) proposed reaction mechanisms for the formation of the fluorescent state of GCaMP-s. In both models Ca^2+^ binding to the N-lobe of CaM is mandatory for the formation of a fluorescent state, whereas C-lobe activation that involves slow Ca^2+^ binding to the C-lobe as the initial step, followed by Ca^2+^ binding to the N-lobe, is only included in a pathway in one of the models (21). However, little is known about the mechanism of RGECIs, and there are no variants with improved kinetic properties available yet.

Following the same design principle that led to GCaMP3_fast_ and GCaMP6f_u_ (23, 24), we introduced specific mutations into jRGECO1a and jRCaMP1a (8) to weaken the interaction between CaM and its target peptide RS20 and to generate faster responding probes. Specific mutations were introduced in the CaM EF hands to disable the Ca^2+^ sites (termed EF-1 to EF-4 according to the mutated hand) and the W4Y mutation in the RS20 target sequence (termed RS-1). The novel variant RGECI probes were analyzed in detail and showed significantly faster transients than their parental variants in ATP-stimulated HEK293T cells. To obtain information about the mechanism of the formation and decay of the highly fluorescent state, the Ca^2+^ response kinetics were analyzed in detail. The results offer a deeper insight into the mechanisms and the effects of the mutations on them for better understanding of GECI and the rational design of new and faster probes.

## Results

Following our hypothesis that weakening the interaction of CaM and RS20 will generate probes with faster kinetics, we disabled single EF hands of CaM by mutating the first conserved aspartate into alanine (25–27) and also altered the binding sequence in RS20 by mutating W9 into Y (RS-1 mutation). In total, nine jRGECO1a and nine jRCaMP1a variants were generated in which the RS-1 mutation and/or one of the four EF-hand mutations were present. The parent and variant proteins were heterologously expressed, purified, and biophysically characterized. Two variants of each jRGECO1a: jRGECO1a EF-1 (f-RGECO1) and jRGECO1a RS-1 EF-4 (f-RGECO2) and jRCaMP1a: jRCaMP1a RS-1 (f-RCaMP1) and jRCaMP1a EF-3 (f-RCaMP2) with the fastest decay were examined in HEK293T cells and hippocampal CA3 neurons for reporting Ca^2+^ dynamics. The kinetics of the fast jRGECO1a and jRCaMP1a variants were determined in detail and where possible an analytical solution was found. The remaining 14 variants are shown in the supporting information (Figs. S2-S9 and Tables S1-S2).

### Fluorescence properties and equilibrium Ca^2+^ binding of jRGECO1a, jRCaMP1a, and their fast-decay variants

In the absorption spectrum the excitation peak for Ca^2+^-dependent fluorescence was ∼570 nm for all RGECIs. For Ca^2+^-bound jRGECO1a and its variants, the peak wavelength was 562 nm with a shoulder at 530 nm. The absorption maximum for Ca^2+^-bound jRCaMP1a and variants lay at 570 nm with a shoulder at 538 nm (Fig. S1). For both jRGECO1a and jRCaMP1a, the peak at ∼570 nm corresponds to the anionic state of the chromophore, more prominent in the presence of Ca^2+^. The absorption peak at 450 nm in the absence of Ca^2+^ is assigned to the neutral state of the chromophore for all RGECIs (28). Most of the fluorescence enhancement for jRGECO1a- and jRCaMP1a-type GECI derived from an increase of the extinction coefficient with fluorescence quantum yield increasing <2-fold by Ca^2+^ binding (Table 1). Fluorescence dynamic ranges (*F*_+Ca_^2+^/*F*_−Ca_^2+^) were determined from three different kinds of measurement: (i) the emission spectra, (ii) Ca^2+^ equilibrium titrations, and (iii) the ratio of brightness values from quantum yield and extinction coefficient measurements, and were in good agreement (Table 1). Taking all these measurements into account, the following average dynamic range values were obtained: 14 ± 3 for jRGECO1a, 10 ± 5 for f-RGECO1, and 14 ± 5 for f-RGECO2. jRCaMP1a and its variants had in general lower dynamic range values with 7 ± 3 for jRCaMP1a, 6 ± 1 for f-RCaMP1, and 6 ± 2 for f-RCaMP2. Thus, the mutations making the probes faster had no adverse effect on the fluorescence dynamic range (Table 2).

**Table 1:**
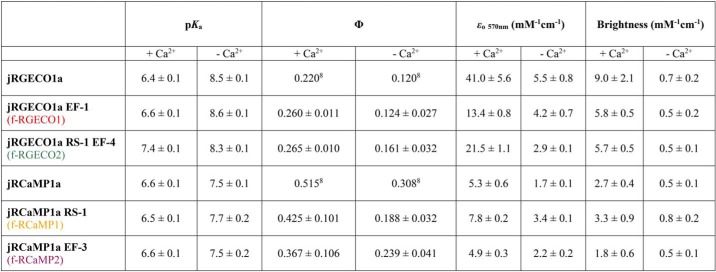
**Summary of *pK_a_*, quantum yield Φ, extinction coefficient ϵ_o_, and brightness of RGECI variants**

**Table 2:**
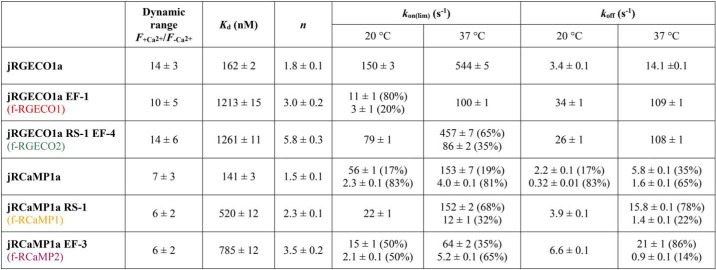
**Dynamic range, *K_d_*, Hill coefficient *n,* and limiting rate constants of RGECI variants**

p*K_a_* values for jRGECO1a and jRCaMP1a and their variants were determined from the pH dependence of the fluorescence intensity in the presence and absence of Ca^2+^ (Figs. S1, *C* and *D*, S2, and S3 and Table S1). For jRGECO1a and f-RGECO1, the p*K_a_* values at saturating Ca^2+^ were 6.4 ± 0.1 and 6.6 ± 0.1. The RS-1 EF-4 mutation shifted the p*K_a_* to 7.4 ± 0.1. In the absence of Ca^2+^, the p*K_a_* values of jRGECO1a and its variants were ∼8.5 ± 0.2. A more detailed look at the pH dependences of jRGECO1a variants in the absence of Ca^2+^ revealed increasing fluorescence intensity with the pH almost reaching the value measured in 1 mm CaCl_2_ at pH 10 (Fig. S2). For the jRCaMP1a variants all Ca^2+^-saturated p*K_a_* values were 6.5 ± 0.2, and in the absence of Ca^2+^ p*K_a_* = 7.5 ± 0.2. In contrast to jRGECO1a, normalized fluorescence for jRCaMP1a and its variants in the absence of Ca^2+^ remained low across the pH range (Fig. S3). The differences in the pH dependences in the absence of Ca^2+^ are thus based on the intrinsic properties of the cp red fluorescent proteins derived from mApple and mRuby (supporting sequence alignment).

**Figure 2.**
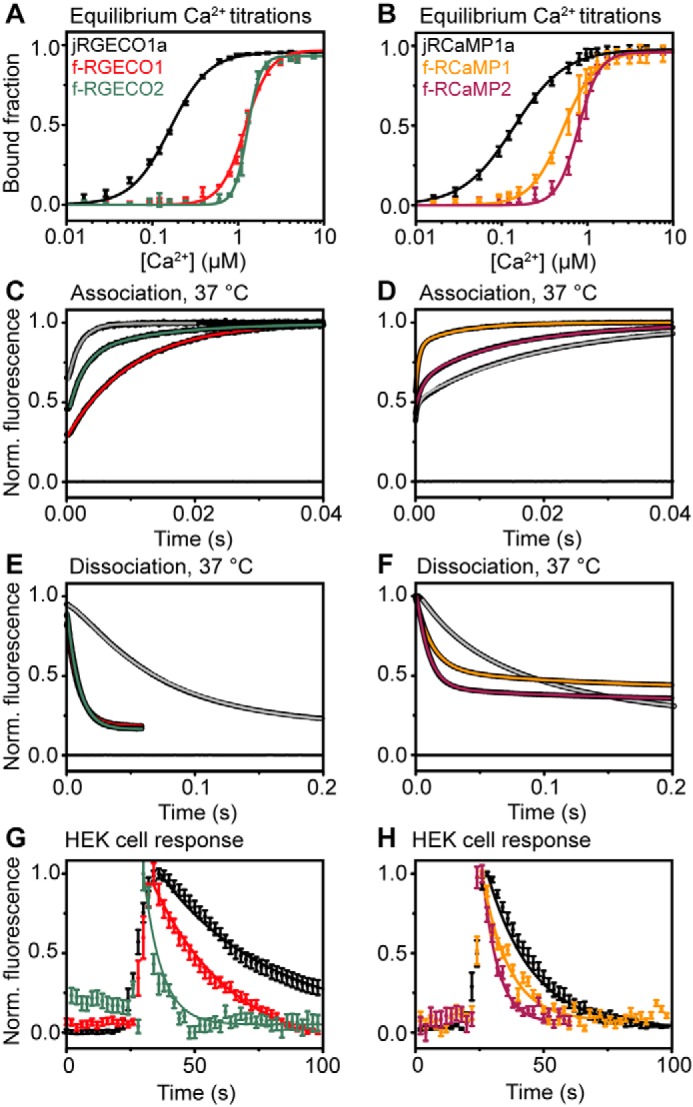
**Biophysical characterization of fast-decay RGECIs.** *A*, equilibrium Ca^2+^ titrations for jRGECO1a (*black squares*) and its fast variants f-RGECO1 (*red squares*) and f-RGECO2 (*green squares*). The data points represent the means with S.D. and are fitted to the Hill equation (*solid lines*). *B*, equilibrium Ca^2+^ titrations for jRCaMP1a (*black squares*) and its fast variants f-RCaMP1 (*yellow squares*) and f-RCaMP2 (*purple squares*). The data points represent the means with S.D. and are fitted to the Hill equation (*solid lines*). *C* and *D*, association kinetics of jRGECO1a (*gray line*), f-RGECO1 (*red line*), and f-RGECO2 (*green line*) (*C*) and of jRCaMP1a (*gray line*), f-RCaMP1 (*yellow line*), and f-RCaMP2 (*purple line*) (*D*) were measured by stopped-flow fluorimetry by mixing the RGECIs in 10 mm EGTA solution with 10 mm CaCl_2_ (concentrations in the mixing chamber) at 37 °C. *E* and *F*, dissociation kinetics of jRGECO1a (*gray line*), f-RGECO1 (*red line*), and f-RGECO2 (*green line*) (*E*) and of jRCaMP1a (*gray line*), f-RCaMP1 (*yellow line*), and f-RCaMP2 (*purple line*) at (*F*) 37 °C. Dissociation kinetics were recorded by rapid mixing of the Ca^2+^ saturated RGECIs with buffer containing a high concentration (12.5 mm in the mixing chamber) of EGTA. The data were normalized to final maximum of 1. Fluorescence recorded when buffer was mixed with buffer containing fluorescent protein is indicated by the line at 0. The data were fitted to either monoexponential or biexponential decays as appropriate. *G* and *H*, Ca^2+^ response kinetics in ATP-stimulated HEK293T cells of jRGECO1a (*black circle*), f-RGECO1 (*red circle*), and f-RGECO2 (*green circle*) (*G*) and of jRCaMP1a (*black circle*), f-RCaMP1 (*yellow circle*), and f-RCaMP2 (*purple circle*) (*H*) with fast variant red GECIs. Ca^2+^ transients were triggered by exposing HEK293T cells to 50 μm ATP. Time courses were recorded at 2-s intervals and are shown for together with their monoexponential decay fit (*solid lines*).

The EF-hand mutations disabled Ca^2+^ binding in one of the binding motifs (25–27), whereas the RS-1 mutation in the RS20 peptide weakened binding. Thus, the mutations were expected to affect the equilibrium dissociation constant, *K_d_*, for Ca^2+^. The dissociation constants of jRGECO1a and jRCaMP1a and their fast-decay variants were determined by titrations in BAPTA (see “Experimental procedures”). jRGECO1a had a high affinity for Ca^2+^ with a *K_d_* of 161 ± 2 nm and a Hill coefficient *n* of 1.8 ± 0.1 measured (Fig. 2*A* and Table 2), similar to the reported values (148 nm *K_d_* and *n* of 1.9 (8)). The *K_d_* values for f-RGECO1 and f-RGECO2 were increased to 1.2 ± 0.2 and 1.3 ± 0.1 μm, respectively, with higher cooperativity shown by Hill coefficients *n* of 3.0 ± 0.2 and 5.8 ± 0.3. jRCaMP1a has been reported to have a strong affinity for Ca^2+^ (*K_d_* of 214 nm) with an essentially linear response to Ca^2+^ (Hill coefficient of 0.9 (8)). In our measurements the binding was even a bit stronger with a *K_d_* of 141 ± 3 nm and also more cooperative with a Hill coefficient of 1.5 ± 0.1 (Fig. 2*B* and Table 2). Both the f-RCaMP1 and f-RCaMP2 mutations slightly lowered the affinity and increased the cooperativity with *K_d_* of 520 ± 12 and 785 ± 12 nm, respectively, and Hill coefficients of 2.3 ± 0.1 and 3.5 ± 0.2. The range of EF-hand and/or peptide mutations thus decreased the affinity between 5- and 10-fold and also increased the cooperativity (Fig. S4).

### Overview of the Ca^2+^ response kinetics of jRGECO1a, jRCaMP1a, and their fast-decay variants

Representative records obtained at saturating [Ca^2+^] (20 μm) at 37 °C show monophasic fluorescence rises with limiting rates (*k*_on(lim)_) of 544 ± 5 s^−1^ for jRGECO1a and 100 ± 1 s^−1^ for f-RGECO1 (Fig. 2*C*). f-RGECO2 had biphasic on-kinetics with *k*_on(lim)_ of 457 ± 7 and 86 ± 2 s^−1^, with relative amplitudes of 65 and 35%, respectively. Thus, the mutations applied did not improve the rise kinetics for f-RGECO1, whereas f-RGECO2 fast phase had similar kinetics to its parental GECI. The dissociation rate for jRGECO1a at 37 °C was 14.1 ± 0.1 s^−1^. f-RGECO1 and f-RGECO2 had 8-fold faster off rates of 109 ± 1 and 108 ± 1 s^−1^, respectively (Fig. 2*E*).

The association kinetics of jRCaMP1a variants were all biphasic at 37 °C, revealing the formation of two fluorescent states (Fig. 2*D*). Rates obtained at saturating [Ca^2+^], *k*_on(lim)_ for jRCaMP1a were 153 ± 7 and 4.0 ± 0.1 s^−1^ with relative amplitudes of 19 and 81%, respectively. f-RCaMP1 showed similar rates of 152 ± 2 s^−1^ and 12 ± 1 s^−1^, but with the faster rate representing the majority of the relative amplitude of 68%. f-RCaMP2 association kinetics were a bit slower with limiting rates of 64 ± 2 and 5.2 ± 0.1 s^−1^ and amplitudes of 35 and 65%, respectively.

The Ca^2+^ off-kinetics of the two new fast variants of jRCaMP1a were biexponential, both showing faster decay rates (*k*_off_) compared with their parental probe. For jRCaMP1a the dissociation rates at 37 °C were 5.8 ± 0.1 and 1.6 ± 0.1 s^−1^ with relative amplitudes of 35 and 65%, respectively (Fig. 2*F*). f-RCaMP1 had a three times faster rate of 15.8 ± 0.1 s^−1^ and a similar slow rate of 1.4 ± 0.1. However, the fast rate represented the majority of the relative amplitude with 78%. For f-RCaMP2 *k*_off_ was 4-fold faster with a value of 21 ± 1 s^−1^ (86%) and a slower rate of 0.9 ± 0.1 s^−1^ (14%).

### Imaging of intracellular Ca^2+^ release in HEK293T cells with fast RGECI

Intracellular Ca^2+^ dynamics and fluorescence dynamic ranges (Δ*F*/*F*_0_) of RGECIs and their fast-decay variants were monitored in ATP-stimulated HEK293T cells. After stimulation the fluorescence rapidly rises and then decays over several seconds. The data were fitted with monoexponential decays resulting in time constants τ of 42 ± 2 s for jRGECO1a, 31 ± 2 s for f-RGECO1, and 5.0 ± 0.5 s f-RGECO2, with Δ*F*/*F*_0_ of 0.81 ± 0.37, 0.59 ± 0.25, and 0.29 ± 0.20, respectively (Fig. 2*G*). For the jRCaMP1a variants the obtained time constants τ were 17.5 ± 0.3 s for jRCaMP1a, 10.4 ± 0.7 s for f-RCaMP1, and 6.0 ± 0.6 s for f-RCaMP2, with Δ*F*/*F*_0_ of 1.00 ± 0.50, 0.42 ± 0.26, and 0.14 ± 0.06, respectively (Fig. 2*H*). Thus, faster response kinetics enabled the RGECI variants to more faithfully report Ca^2+^ dynamics in the cells.

### Imaging of Ca^2+^ transients in hippocampal slices

**Figure 3.**
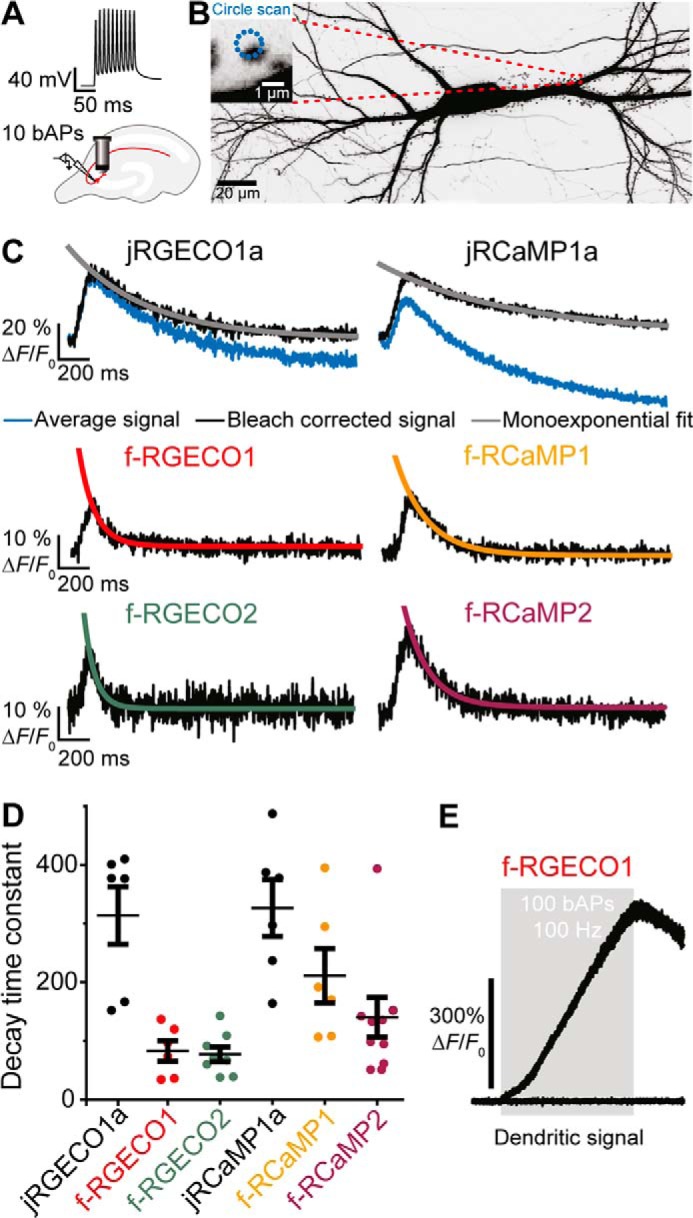
**Spine Ca^2+^ transients in response to backpropagating action potentials.** *A*, backpropagating action potentials are elicited in a transfected CA3 pyramidal cell by somatic current injections. Ca^2+^ transients are simultaneously optically recorded from a single spine with two-photon imaging. *B*, maximum intensity projection of two-photon image stacks of CA3 pyramidal neuron expressing jRGECO1a and GFP, 8 days after electroporation. Green fluorescence intensity is shown as inverted gray values. The *scale bar* represents 20 μm (neuron) and 1 μm (single spine). *C*, decay time measurements after bleaching correction (*red trace*) for individual experiments (five trials averaged per spine) by single exponential fit. *D*, decay time constants measured in individual spines in response to 10 backpropagating action potentials of CA3 neurons expressing jRGECO1a (τ_off_ = 314 ± 49 ms; *n* = 6 spines); f-RGECO1 (τ_off_ = 83 ± 17 ms; *n* = 6 spines); f-RGECO2 (τ_off_ = 77 ± 13 ms; *n* = 8 spines); jRCaMP1a (τ_off_ = 327 ± 49 ms; *n* = 6 spines); f-RCaMP1 (τ_off_ = 211 ± 46 ms; *n* = 6 spines); and f-RCaMP2 (τ_off_ = 140 ± 34 ms; *n* = 9 spines). The values are expressed as means ± S.E. *E*, dendritic Ca^2+^ transients in response to 100 backpropagating action potentials at 100 Hz (train duration, 1 s; *gray box*) measured in the apical dendrite of a CA3 neuron expressing f-RGECO1. The low affinity of f-RGECO1 prevents the signal from reaching a saturating plateau even in response to a train of 100 backpropagating action potentials.

The novel fast variants were expressed in CA3 pyramidal neurons of hippocampal slices. Backpropagating action potentials (bAP) were induced by somatic current injections and the Ca^2+^ transients simultaneously optically recorded from a single spine with two-photon imaging (Fig. 3, *A* and *B*). The signals in response to 10 bAPs at 100 Hz from six to nine spines were averaged and corrected for bleaching. By fitting these signals with monoexponential decays the following time constants (τ_off_) were obtained: jRGECO1a 314 ± 49 ms, f-RGECO1 83 ± 17 ms, f-RGECO2 77 ± 13 ms, jRCaMP1a 327 ± 49 ms, f-RCaMP1 211 ± 46 ms, and f-RCaMP2 140 ± 34 ms (Fig. 3, *C* and *D*). Thus, the novel RGECI variants showed up to 4-fold faster off rates compared with their parental sensors with comparable dynamic ranges. It must be noted that in dendritic spines, because of their lower affinities (*K_d_* = ∼5 times higher), the signal amplitudes and signal to noise ratios are lower for the fast sensors than for the parent probes (Fig. S9). The advantage of the fast probes is, however, evident when monitoring Ca^2+^ transients in the dendrites, where the f-RGECO1 fluorescence on-response to 100 bAPs displayed linearity, indicating that because of the variant's reduced Ca^2+^ affinity, the saturation commonly observed with higher affinity sensors did not occur with f-RGECO1 (Fig. 3*E*). It should be noted that RGECIs based on mApple, like jREGECO1a and its variants, showed strong photoswitching as already reported in earlier studies (8). Thus, these indicators showed a fluorescence response induced by the near IR excited light, which needs to be included to correctly analyze their signal.

### Kinetic mechanisms of RGECI and fast-decay variants

The above described association kinetics at 37 °C were obtained at saturating [Ca^2+^]. The observed association rate, *k*_obs_, was, however, dependent on [Ca^2+^]. When *k*_obs_ was plotted against [Ca^2+^], a variety of patterns were observed. Kinetic models were developed where possible and are introduced through the highlighted fast variants with further examples shown in the supporting information (Figs. S5-S8). Three types of behavior are described using the fast-decay variants as class leading examples.

### jRGECO1a

**Figure 4.**
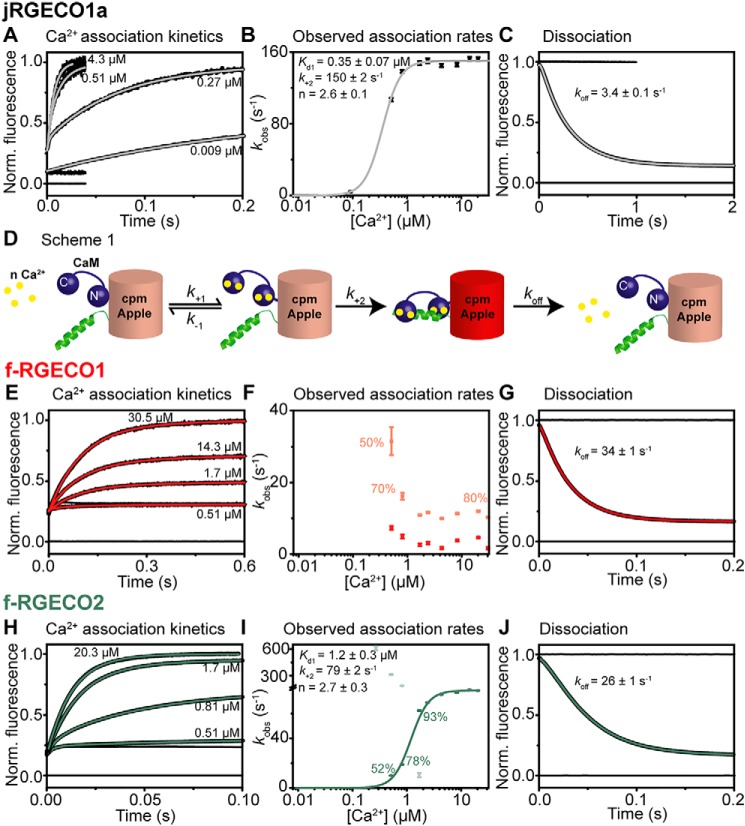
**Kinetic mechanism of jRGECO1a and its fast variants.** The data were collected at 20 °C. *A*, *E*, and *H*, Ca^2+^ association kinetics of jRGECO1a (*A*), f-RGECO1 (*E*), and f-RGECO2 (*H*). Records in order of increasing amplitude are as follows: buffer mixed with buffer (zero line), buffer mixed with jRGECI (10 mm EGTA in mixing chamber), buffers with the indicated final [Ca^2+^] (in the mixing chamber) mixed with jRGECI (10 mm EGTA in mixing chamber). *B*, *F*, and *I*, plots of [Ca^2+^] dependence of observed association rates of jRGECO1a (*B*), f-RGECO1 (*F*), and f-RGECO2 (*I*). The sigmoidal curves represent the best fit to Equation 1 derived for Scheme S1. *D*, the fitted parameters are shown in the panels. *C*, *G*, and *J*, Ca^2+^ dissociation kinetics of jRGECO1a (*C*), f-RGECO1 (*G*), and f-RGECO2 (*J*). Record at 0 represents trace when buffer was mixed with buffer (zero line), time trace at 1 corresponds to buffer mixed with jRGECI (1 mm Ca^2+^ in mixing chamber), whereas the decays were recorded when jRGECIs were mixed with EGTA (12.5 mm EGTA in mixing chamber).

Association kinetic records were monoexponential (Fig. 4*A*), and the observed association rates, *k*_obs_, were plotted as a function of [Ca^2+^] (Fig. 4*B*). At low [Ca^2+^] *k*_obs_, tended to 0 and then, showing a cooperative pattern, saturated as a function of [Ca^2+^]. The simplest model to fit the data depicts a two-step process in which rapid, cooperative Ca^2+^ binding is followed by isomerization involving the binding of the RS20 peptide to Ca^2+^*_n_*.CaM leading to the development of the highly fluorescent state (indicated by the *red barrel* in Fig. 4*D*; Scheme S1). The reversal of the process, peptide dissociation from the Ca^2+^-bound complex would be extremely slow. However, an alternative dissociation pathway allows rapid decay via Ca^2+^ dissociation followed by peptide dissociation. The on-reaction therefore can be treated kinetically as an essentially irreversible process. The plot of *k*_obs_ against [Ca^2+^] is fitted to Equation 1 with an *K_d_*_(overall)_ (Equation 2).

(Eq. 1) kobs=k+2[Ca2+]nKd1+n[Ca2+]n

(Eq. 2) Kd(overall)=Kd1+nk+2k+1

From the fit *K_d_*_1_ of 0.35 ± 0.07 μm, *k*_+2_ of 150 ± 2 s^−1^ and *n* of 2.6 ± 0.1 were derived (Fig. 4*B*). The fitted *K_d_*_1_ value was similar to the measured *K_d_* (162 ± 2 nm); therefore it is assumed that Ca^2+^ and jRGECO1a are in a rapid equilibrium (*k*_−1_ ≫ *k*_+2_), with *k*_+1_ probably close to diffusion limited (∼10^8^ m^−1^ s^−1^). On Ca^2+^ sequestration, the fluorescent state decays via a different pathway from that of its formation: Ca^2+^ dissociation from the fluorescent complex is followed by the weak peptide complex rapidly falling apart. The measured dissociation rate for jRGECO1a was 3.4 ± 0.1 s^−1^ (Fig. 4*C*). Similar patterns were observed for jRGECO1a EF-3, jRGECO1a EF-4, and jRGECO1a RS-1 EF-3 variants (Fig. S5). jRGECO1a RS-1 EF-1 (Fig. S5*C*) had a limiting association rate (*k*_on(lim)_) of 23 ± 1 s^−1^ and a dissociation rate (*k*_off_) of 64 ± 1 s^−1^ (Table S1). Their Ca^2+^-dependent observed association rate showed an all-or-none pattern. Their mechanisms may be consistent with Scheme S1, with the amplitudes at low [Ca^2+^] too small to detect.

### Fast decay variant f-RGECO1

Association kinetic records were biexponential (Fig. 4*E*), and the observed association rates, *k*_obs_, were plotted as a function of [Ca^2+^] (Fig. 4*F*). Below 0.51 μm free [Ca^2+^], no fluorescence increase was observed. In contrast to jRGECO1a, both association rates decrease with increasing [Ca^2+^], suggesting a mechanism in which a slow pre-equilibrium precedes Ca^2+^ binding. Two forms of the apo state, with only one binding Ca^2+^, could be in equilibrium. The biphasic behavior may indicate two independent populations of the variant. However, dissociation was monoexponential with a rate of 34 ± 1 s^−1^ (Fig. 4*G*).

### Fast-decay variant f-RGECO2

A more complex underlying mechanism was observed for fast-decay variant f-RGECO2 (Fig. 4, *H* and *I*). Initially, at [Ca^2+^] lower than 1 μm, a biphasic process was observed, indicating the existence of two fluorescent intermediates. The amplitude of the faster rate decreased with increasing [Ca^2+^], and at [Ca^2+^] > 2 μm, a single phase was observed apparently at the saturating rate of the slow phase (*k*_on(lim)_) of 79 ± 1 s^−1^. We attribute the fast phase to CaM N-lobe Ca^2+^ binding leading to a fluorescent state. The plot of the second, slow phase was fitted to Scheme S1, giving *K_d_*_1_ of 1.2 ± 0.1 μm, *k*_+2_ of 79 ± 2 s^−1^ and *n* of 2.7 ± 0.3. The fitted *K_d_*_1_ of the slow phase is in good agreement with the measured *K_d_* of 1.26 ± 0.02 μm.

jRGECO1a RS-1, jRGECO1a EF-2, and jRGECO1a RS-1 EF-2 (Fig. S6) showed a similar behavior with a bell-shaped appearance of *k*_obs_ for a fast phase as a function of [Ca^2+^] and a sigmoidal saturating curve for the slow phase (Fig. S6, *A* and *B*). In contrast, the fast and slow phases for jRGECO1a RS-1 EF-2 had different saturation values at high [Ca^2+^], suggesting two final fluorescent states (Fig. S6*C*). For jRGECO1a RS-1 and jRGECO1a RS-1 EF-2, the fitted *K_d_*_1_ values of the fast phase were in the same range as their measured *K_d_* values, 428 ± 4 and 429 ± 5 nm, respectively. However, for jRGECO1a EF-2, they are in less good agreement (*K_d_*_1_ of 1.1 ± 0.2 μm *versus K_d_* of 436 ± 5 nm), indicating that the fast phase has a bigger impact and cannot be neglected for the estimate.

### jRCaMP1a

**Figure 5.**
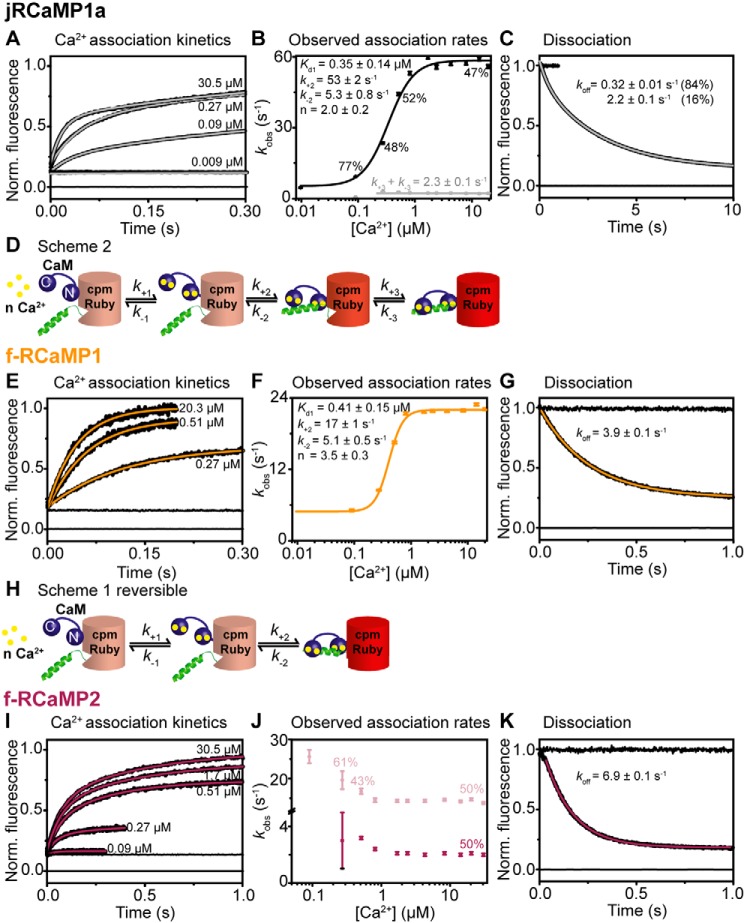
**Kinetic mechanism of jRCaMP1a and its fast variants.** The data were collected at 20 °C. *A*, *E*, and *I*, Ca^2+^ association kinetics for jRCaMP1a (*A*), f-RCaMP1 (*E*), and f-RCaMP2 (*I*). Records in order of increasing amplitude are buffer mixed with buffer (zero line), buffer mixed with jRGECI (10 mm EGTA in mixing chamber), and buffers with the indicated final [Ca^2+^] (in the mixing chamber) mixed with jRGECI (10 mm EGTA in mixing chamber). *B*, *F*, and *J*, plots of [Ca^2+^] dependence of observed association rates for jRCaMP1a (*B*), f-RCaMP1 (*F*), and f-RCaMP2 (*J*). The sigmoidal curve represents the best fit to Equation 3 derived from Scheme S1. *H*, the fitted parameters are shown in the panel. *D*, jRCaMP1a shows two fluorescent states and thus follows a more complex mechanism (Scheme S2). *C*, *G*, and *K*, Ca^2+^ dissociation kinetics of jRCaMP1a (*C*), f-RCaMP1 (*G*), and f-RCaMP2 (*K*). Record at 0 represents trace when buffer was mixed with buffer (zero line), time trace at 1 corresponds to buffer mixed with jRGECI (1 mm Ca^2+^ in mixing chamber), whereas the decays were recorded when jRGECIs were mixed with EGTA (12.5 mm EGTA in mixing chamber).

The Ca^2+^ dependence of the observed on-kinetics of jRCaMP1a followed a complex pattern with a fast, Ca^2+^-dependent saturating and a slow, Ca^2+^-independent rate (Fig. 5, *A* and *B*). The two phases presented with equal amplitudes. This behavior can be explained with a mechanism in which binding is followed by two reversible isomerizations with two fluorescent states (Fig. 5*D* and Scheme S2). The plot of the fast rate, *k*_obs1_, against [Ca^2+^] is fitted to Equation 3 derived from Scheme S1 with a reversible second step, whereas the Ca^2+^-independent slow rate, *k*_obs2_, represents the sum of the rate constants for the second isomerization (Equation 4).

(Eq. 3) kobs1=k+2[Ca2+]nKd1+n[Ca2+]n+k−2

(Eq. 4) $$k_{\text{obs}2}=k_{+3}+k_{-3}$$

(Eq. 5) Kd(overall)=nKd1n1+K2+K2K3

The fit to the fast phase gave a *K_d_*_1_ of 0.35 ± 0.14 μm, a *k*_+2_ of 53 ± 2 s^−1^, a *k*_−2_ of 5.3 ± 0.8 s^−1^, and *n* of 2.0 ± 0.2. For *k*_+3_ + *k*_−3_, 2.3 ± 0.1 s^−1^ was obtained. If we assume that *K*_2_*K*_3_ is negligible a *K_d_*_(overall)_ of 105 ± 34 nm is calculated, which is in good agreement with the measured one (*K_d_* = 141 ± 3 nm). The Ca^2+^ off-kinetics of jRCaMP1a were biexponential, with rates of 2.2 ± 0.1 and 0.32 ± 0.01 s^−1^ and relative amplitudes of 17 and 83%, respectively, consistent with two successive reversible steps. Similar behavior was observed for jRCaMP1a EF-1, jRCaMP1a EF-2, and jRCaMP1a EF-4 (Fig. S8).

### Fast-decay variant f-RCaMP1

The Ca^2+^ dependence of the observed association rate of f-RCaMP1 showed a sigmoidal pattern; however, in contrast to jRGECO1a, the rates at low [Ca^2+^] did not tend to 0 but to a finite minimum rate (Fig. 5, *E* and *F*). The observed association rates saturated at 22 ± 1 s^−1^, and the intercept tends to 5.1 ± 0.5 s^−1^. This behavior is consistent with a two-step mechanism in which cooperative Ca^2+^ binding is followed by a reversible isomerization (Scheme S1 and Fig. 5*H*) and the intercept corresponds to *k*_-2_. Fluorescence develops in the second step. *k*_obs_ is expressed in Equation 3, and the system is constrained by *K_d_*_(overall)_ (Equation 6).

(Eq. 6) Kd(overall)=nKd1n1+K2

Using Equation 3 to fit the data, the two equilibrium constants *K_d_*_1_ of 0.41 ± 0.15 μm^−1^ and *K*_2_ of 3.3 ± 0.6 gave an *K_d_*_(overall)_ of 0.27 ± 0.08 μm, which is close to the measured value of 0.52 ± 0.02 μm (Table 2). In terms of dissociation of the Ca^2+^-saturated fluorescent complex, peptide dissociation initiates the reversal from the fluorescent state with a measured off rate of 3.9 s^−1^ (Fig. 5*G*). Variants jRCaMP1a RS-1 EF-1 and jRCaMP1a RS-1 EF-2 also fit well to Scheme S1, with the fitted *K_d_*_(overall)_ (250 and 291 nm) a bit smaller than the measured ones (702 and 607 nm) (Fig. S7, *A* and *B*, and Table S3).

Variants jRCaMP1a RS-1 EF-3 and jRCaMP1a RS-1 EF-4 show an all-or-none pattern of Ca^2+^-dependent association rates, with biphasic dissociation kinetics (Fig. S7, *C* and *D*). The mechanism for these variants is too complex for an analytical solution.

### Fast-decay variant f-RCaMP2

Fast variant f-RCaMP2 is characterized by two fluorescent states of equal amplitude with saturating rates of 15 ± 1 and 2.1 ± 0.1 s^−1^ (Fig. 5, *I* and *J*). For both, *k*_obs_ decreases with increasing [Ca^2+^], similar to f-RGECO1. Thus, there is a slow pre-equilibrium preceding Ca^2+^ binding with two forms of the apo state, with only one binding Ca^2+^, that could be in equilibrium. The biphasic behavior may indicate two independent populations of the variant. However, dissociation was monoexponential with a rate of 6.6 ± 0.1 s^−1^ (Fig. 5*K*).

## Discussion

We generated fast-decay variants of jRGECO1a and jRCaMP1a using the rational design strategy previously applied to GCaMPs (23, 24). In HEK293T cells, fast-decay variants f-RGECO1 and f-RGECO2 showed 1.4- and 8-fold, and f-RCaMP1 and f-RCaMP2 had 1.7- and ∼3-fold faster decay kinetics than their respective parent proteins, indicating the utility of fast-responding probes, *e.g.* f-RGECO2 in faithful monitoring of even slow intracellular Ca^2+^ dynamics.

The advantage of the fast response kinetics of the probes is clearly seen in hippocampal CA3 pyramidal cells, in which rapid Ca^2+^ transients are used to visualize action potential firing. 10 bAPs were detected by our fast variants, with an up to 4-fold faster decay rate (τ_off_ of 77 ms for f-RGECO2) compared with jRGECO1a. Fast jRCaMP1a variants f-RCaMP1 and f-RCaMP2 showed similar improvements in the decay kinetics with time constants of 211 and 140 ms, respectively, with the added advantage of not showing the photoswitching characteristic of jRGECO1a. Moreover, it should be noted that the *in situ* decay rates were ∼10-fold slower than those measured in solution, indicating that the probe kinetics are not limiting the detection of the Ca^2+^ transient.

The main absorption peaks at 562 and 570 nm for jRGECO1a and jRCaMP1a, respectively, correspond to the anionic state of the chromophore and diminish in the absence of Ca^2+^ consistent with the equilibrium of the chromophore shifting to the protonated form. A small shift of 4 nm seen in the Ca^2+^-free state for jRGECO1a and jRCaMP1a to 566 and 574 nm, respectively, is similar to that observed for GCaMP variants and is caused by a change in the local electric field at the chromophore caused by Ca^2+^-dependent rearrangements of the surrounding residues (28). The absorption peak at 450 nm in the absence of Ca^2+^ is assigned to the neutral state of the chromophore for all RGECIs (28). This peak is more prominent in the spectra of jRGECO1a than for jRCaMP1a relative to the 574- and 542-nm peaks, respectively, indicating that the equilibrium between the protonated and deprotonated forms of the chromophore is differently poised for the two types of red-fluorescent core proteins, contributing to the greater fluorescence dynamic range of jRGECO1a and variants compared with jRCaMP1a.

The hypothesis that weakening the Ca^2+^/CaM–RS20 peptide interaction by EF-hand and peptide mutations will positively affect the decay kinetics has proven correct overall. The EF-hand and/or peptide mutations, previously found to affect the affinity of GCaMP-s for Ca^2+^, only slightly affected the Ca^2+^ affinity of the jRGECO1a and jRCaMP1a variants. For jRGECO1a most variants were shifted from ∼150 to ∼450 nm, whereas the variants with the fastest dissociation kinetics had *K_d_* values of ∼1 μm. jRCaMP1a variants were either not affected at all (EF-1 and EF-2) or had slightly lower affinities, with *K_d_* values of ∼700 nm. However, comparing the specific site mutation combinations best improving the kinetics and the fluorescence brightness and dynamic range reveals that interactions with the fluorescent proteins affect the properties of the Ca^2+^/CaM–RS20 peptide complex. For jRGECO1a mutation of the EF1 hand leads to faster Ca^2+^ dissociation rates but slows down the limiting association rates significantly (f-RGECO1 and jRGECO1a RS-1 EF-1). Although the mutation of the EF3/EF4 hands and RS-1 alone had little effect on the association and dissociation kinetics, the combination of RS-1 and EF-4 (f-RGECO2) generated a probe with ∼8-fold accelerated Ca^2+^ dissociation kinetics. For jRCaMP1a the EF-3, EF-4 and RS-1 mutation and the combination of them generated the fastest probes with up to 13-fold faster decay kinetics. This behavior resembles GCaMP3 and GCaMP6f with the combination of RS-1 and EF-3 mutations generating the fastest variants (23, 24). However, for GCaMP3 and GCaMP6f the mutation that had the most significant influence on the kinetics also decreased the dynamic range most drastically. This also holds true for the fastest variants of jRGECO1a and jRCaMP1a, with jRGECO1a RS-1 EF-1 mutation causing a 50% decrease in the brightness and dynamic range, whereas in jRCaMP1a the EF-4, RS-1 EF-3, and RS-1 EF-4 mutations diminished the dynamic range to the greatest extent (1.4–2.2).

In this study the Ca^2+^ dependence of the on-response for all variants was extensively studied, giving insight into the variety of association kinetics. jRGECO1a followed a simple dependence that could be fitted to a two-step model in which pre-equilibrium Ca^2+^ binding is followed by a rate-limiting conformational change to form the fluorescent state. Some variants (jRGECO1a RS-1, jRGECO1a EF-2, jRGECO1a RS-1 EF-2, and f-RGECO2) showed a fast phase with a bell-shaped dependence on Ca^2+^ concentration and a slow phase the rate of which tended to 0, indicating that the dissociation process occurs by a different pathway from the association, by Ca^2^ dissociation followed by the peptide (Fig. S5). It was thus hypothesized that two fluorescent states were formed in parallel processes. The fast phase could correspond to the Ca^2+^-bound CaM N-lobe forming a fluorescent complex with the peptide. This complex then would be able to bind two more Ca^2+^ at the CaM C-lobe. In parallel, the fully Ca^2+^-bound CaM forms a peptide complex at the slower rate determined by the slower C-lobe Ca^2+^ binding (29). At lower [Ca^2+^] the greater association rate constant drives the process to the partially Ca^2+^-saturated complex, whereas at higher [Ca^2+^] the fully cooperative binding sequence involving the C-lobe dominates (jRGECO1a RS-1, jRGECO1a EF-2, and jRGECO1a RS-1 EF-4). This finding is in agreement with previously proposed models, where a partially bound fluorescent complex (Ca^2+^_2N_.CaM.RS20*) was predicted, with the possibility of binding two further Ca^2+^ to the C-lobe to generate the fully bound and highly fluorescent complex (Ca^2+^_4_·CaM.RS20*) (21, 24). The model of Sun *et al.* (21) suggests a C-lobe activation that involves slow Ca^2+^ binding to the C-lobe as the initial step, followed by Ca^2+^ binding to the N-lobe that leads to the formation of the fluorescent complex. In contrast, in the model of Helassa *et al.* (24), the fast binding to the N-lobe is always the initial step. jRGECO1a is different from GCaMP3 and GCaMP6s/m/f because the Ca^2+^ binding to the N-lobe is not mandatory for the formation of a fluorescent state. For the red GECIs, formation of a CaM C-lobe complex with the peptide is sufficient for the formation of a fluorescent state.

In contrast to the jRGECO1a variants with bell-shaped Ca^2+^ plots, jRGECO1a RS-1 EF-2 shows two fluorescent states at high [Ca^2+^]; thus when the EF-2 and RS-1 mutations are combined, the three-Ca^2+^ ion-bound state is not the dominant species, and both fluorescent states co-exist. Ca^2+^ dissociation from the Ca^2+^-saturated state occurs by a rapid single exponential process consistent with Ca^2+^ coming off leading the dissociation process (Fig. S7*C*).

jRCaMP1a and its variants mainly showed two different kinds of pattern in terms of the [Ca^2+^] dependence of the observed association rates. The parental variant and all others that do not contain the RS-1 mutation had two phases, with the fast one following a sigmoidal saturating Ca^2+^ dependence and the slow one Ca^2+^-independent, suggesting the formation of two fluorescent states, which show biexponential dissociation kinetics. In contrast, all jRCaMP1a variants carrying the RS-1 mutation seem to miss the Ca^2+^-independent slow rate. However, measurements at 37 °C for f-RCaMP1 revealed a slow phase with small amplitude. Furthermore jRCaMP1a RS-1 EF-3 and jRCaMP1a RS-1 EF-4 show a biexponential dissociation like their parental GECI. These results indicate that the weakening of the interaction of the CaM C-lobe with the N terminus of the RS20 peptide intensively slows down the second isomerization until it becomes undetectable at 20 °C. Although this model explains the observed kinetics of almost all jRCaMP1a variants and the influence of the RS-1 mutation, f-RCaMP2 and f-RGECO1 seem to follow their own specific pattern. Both observed rates decrease slightly with increasing [Ca^2+^] and are almost [Ca^2+^]-independent. Such behavior may be explained with a model involving two Ca^2+^-free states that are in equilibrium with each other, with only one able to bind Ca^2+^. Thus, the association rates would not increase with increasing [Ca^2+^] but are rather entirely dependent on this pre-equilibrium. This model describes similar kinetics observed for enzymes binding to ligands, that have an open (ligand can bind) and closed (ligand cannot bind) conformation (30). However, deriving a simple fitting equation for such a model containing only a few parameters was not possible.

The variety of dependences of the association rates on the [Ca^2+^] shows that the response kinetics are not solely dependent on the Ca^2+^*_n_*·CaM–RS20 peptide interaction but on more intricate interactions with the chromophore-containing β-barrel; therefore the limiting isomerization rate may correspond to either the Ca^2+^*_n_*·CaM–RS20 interaction or the subsequent β-barrel closure and stabilization, followed by rapid deprotonation. Comparison of the crystal structures shows that RGECO1 (PDB code 4I2Y (7)) is much more similar to GCaMPs (PDB code 3WLD (31)) with regard to the length of the RS20 peptide helix with the Ca^2+^*_n_*·CaM–RS20 complex positioned right in front of the opening of the distorted β-barrel. Between RGECO1 and RCaMP (PDB code 3U0K (7)), the helix points into different directions (up in RGECO1 and down in RCaMP).

Our study showed that the strategy of weakening the interaction of the RS20 peptide and CaM generates faster probes of the red fluorescent protein–based genetically encoded Ca^2+^ indicators, similarly to GCaMPs. However, RGECI variants differ from GCaMPs in their mechanism with the N-lobe binding as a required step for fluorescence increase. Furthermore, the red GECIs show a far more complex variety of kinetic responses, which were explained by and fitted to simple models. jRGECO1a and jRCaMP1a are able to detect a single action potential *in vivo* (10, 32). In contrast the novel fast variants presented in this study will be superior for *in vivo* imaging of fast, high-amplitude signals, *e.g.* during release from intracellular Ca^2+^ stores or dendritic Ca^2+^ waves. The new indicators will report such events with excellent linearity and in unfiltered kinetic detail. Thus, our new sensors will be excellent for applications in *in vivo* imaging of muscle contraction and high firing neurons.

## Experimental procedures

### Site-directed mutagenesis

The genes of the parental GECIs pGP-CMV-NES-jRCaMP1a and pGP-CMV-NES-jRGECO1a were a gift from Douglas Kim (Addgene plasmid nos. 61562 and 61563) (8). jRCaMP1a and jRGECO1a genes were amplified by PCR using the following primers (NdeI-fw and NotI-rv) and subcloned into pET30b by restriction ligation using NdeI/NotI and T4 DNA ligase: NdeI-fw, GGAATCCATATGCTGCAGAACGAGC; and NotI-rv, GGTGCTCGAGTGCGGCCGCCTA.

The pET30b-jRCaMP1a and pET30b-jRGECO1a plasmids were used for heterologous expression in *Escherichia coli*. Mutations in the EF hands and the RS20 peptide were performed using the QuikChange XL mutagenesis protocol using the following 5′ to 3′ primers: for jRCaMP1a and jRGECO1a: RS-1 W44Y, CGACTCATCACGTCGTAAGTACAATAAGGCAGGTCACGCAG; EF-1 D323A, GCTTTCTCCCTATTTGCCAAGGACGGGGATGGG; EF-2 D359A, CATGATCAATGAAGTAGCTGCCGACGGTGACGGC; and only for jRGECO1a: EF-3 D396A, GCGTTCCGCGTGTTTGCTAAGGACGGCAATGGC; EF-4 D432A, GATCAGGGTAGCAGCCATCGATGGGGATGG; and only for jRCaMP1a: EF-3 D396A, GCGTTCGGCGTGTTTGCTAAGGATGGCAATGGC; EF-4 D432A, GATCAGGGAAGCAGCCAGCGATGGGGATGG. Mutations were confirmed by DNA sequencing (Genewiz®).

### Protein expression and purification

Proteins were expressed using *E. coli* BL21 (DE3) gold cells. Cells were grown at 37 °C, and protein expression was induced at *A*_600_ of 0.8 with 0.4 mm isopropyl β-d-1-thiogalactopyranoside overnight at 20 °C. The cells were harvested, resuspended in 50 mm Na^+^-HEPES, 200 mm NaCl, pH 7.5, containing protease inhibitors (EDTA-free Complete®, Fisher Thermo Scientific). The cells were lysed by sonication on ice (2- and 8-s pauses, intensity 55%, 2 min of total time). Protein was purified using a HisTrap column with an Äkta Explorer system (GE Healthcare). Protein was eluted in 50 mm Na^+^-HEPES, 200 mm NaCl, pH 7.5, containing 500 mm imidazole. Fractions containing the protein were dialyzed overnight against storage buffer (50 mm K^+^-HEPES, 100 mm KCl, pH 7.5) at 4 °C. Purity was checked by SDS-PAGE (∼90% pure), and aliquots were stored at −80 °C. Protein concentrations were determined spectroscopically using extinction coefficients calculated from their amino acid sequence (33) (jRGECO1a and all variants with EF mutation ϵ_0(280 nm)_ = 40,680 m^−1^ cm^−1^, all jRGECO1a variants containing RS-1 mutation ϵ_0(280 nm)_ = 36,270 m^−1^ cm^−1^, jRCaMP1a and all EF mutants ϵ_0(280 nm)_ = 32,430 m^−1^ cm^−1^, all jRCaMP1a variants containing RS-1 mutation ϵ_0(280 nm)_ = 28,020 m^−1^ cm^−1^).

### pH titrations

To determine the p*K_a_* of the red GECIs fluorescence emission spectra (λ_ex_ = 570 nm, λ_em_ = 573–630 nm) at different pH levels were recorded at a Fluorolog3 (Horiba©). Buffer solutions with 50 mm buffer (K^+^-MES, pH 6; K^+^-HEPES, pH 7–8; Tris-HCl, pH 8.5–9; CHAPS-HCl, pH 10), 100 mm KCl, and 2 mm MgCl_2_ containing either 1 mm CaCl_2_ or 2 mm BAPTA were prepared and protein to a final concentration of 1 μm was added. BAPTA was chosen as Ca^2+^ chelator because of its stable Ca^2+^ affinity over a wide pH range.

### Quantum yield, extinction coefficients, and brightness determination

The fluorescence quantum yield (Φ) was determined relative to the parental red GECIs jRCaMP1a and jRGECO1a (8). The absorption spectra of red GECIs in assay buffer (50 mm K^+^-HEPES, 100 mm KCl, 2 mm MgCl_2_ pH 7.5) containing either 1 mm CaCl_2_ or 2 mm EGTA were measured with a Lambda Bio40 (PerkinElmer Life Sciences) and the fluorescence emission spectra (λ_ex_ = 570 nm, λ_em_ = 573–650 nm) were recorded at a Fluorolog3 (Horiba). The maximum absorption was plotted against the integrated fluorescence, and the points were fitted with a linear regression line. The quantum yield of the variants was calculated by using the slope and the reported quantum yield of the parental GECIs: jRGECO1a Φ(+Ca^2+^) = 0.220, Φ(−Ca^2+^) = 0.120; RCaMP1a Φ(+Ca^2+^) = 0.515, Φ(−Ca^2+^) = 0.308 (8) (Φ_variant_ = slope_variant_/slope_ref_ * Φ_ref_; Equation 8). Extinction coefficients ϵ_0_ at 570 nm were determined using the protein concentration calculated from the absorption at 280 nm and the ϵ_0(280 nm)_ values (see “Protein purification and expression” above). The brightness was calculated by multiplying the extinction coefficient by the quantum yield.

### Equilibrium Ca^2+^ titrations

The dissociation constant (*K_d_*) values were determined by following the change in fluorescence emission (λ_ex_ = 570 nm; λ_em_ = 592 nm) on a Fluorolog3 (Horiba) with increasing Ca^2+^ concentrations. Ca^2+^ solution (325 mm) was titrated using an ALLADIN syringe pump (10 μl/min) into the protein solution in assay buffer containing 5 mm BAPTA, pH 7.5, while constantly stirring. The fluorescence was corrected for the dilution, and the free [Ca^2+^] was calculated using MaxChelator® software. The data were fitted with the Hill equation for specific binding using GraphPad Prism 6 (*y* = *B*_max_*x^n^/(*K_d_^n^* + *x^n^*)).

### Fluorescence dynamic range determination

The fluorescence dynamic range *F*_+Ca_^2+^/*F*_−Ca_^2+^ was determined as the average of values obtained from different measurements: (i) from the maxima of the spectra obtained in the pH titrations in assay buffer with 1 mm CaCl_2_ or 2 mm BAPTA, (ii) calculated from the ratio of brightness obtained from the quantum yield measurements, and (iii) the ratio of the end point (1 mm [Ca^2+^]) and the starting fluorescence values (5 mm EGTA) in the titration experiments.

### Stopped-flow kinetics

A Hi-Tech Scientific KinetAsyst^TM^ double-mixing stopped-flow apparatus was used in single mixing mode. Fluorescence was excited at 577 nm, and emission was detected using a long pass filter (>590 nm). For Ca^2+^ association kinetics, RGECI (concentration in the 0.1–1 μm range) in assay buffer (50 mm K^+^-HEPES, 100 mm KCl, 2 mm MgCl_2_, pH 7.5) containing 10 mm EGTA was rapidly mixed with assay buffer containing increasing concentration of [Ca^2+^]. For dissociation kinetics, RGECI in assay buffer containing 0.5 mm CaCl_2_ was rapidly mixed with assay buffer containing 12.5 mm EGTA (concentrations in mixing chamber). [Ca^2+^] concentrations were calculated using MaxChelator® software. The data were fitted either with a monoexponential or a biexponential function.

### Imaging of RGECI variants in HEK293T cells

The cells were cultured at 37 °C and 5% CO_2_ in a black 24-well glass-bottomed plate in Dulbecco's modified Eagle's medium containing nonessential amino acids (Life Technologies), 10% heat-inactivated fetal bovine serum (Life Technologies) and penicillin/streptomycin (100 units/ml, 100 mg/ml, respectively). Transfection of cells was carried out with 0.5 μg of DNA and 2.5 μl of Lipofectamine 2000 in 100 μl of OptiMem® for 16–48 h at 37 °C. The cells were imaged 16–48 h post-transfection in 2 or 1 ml of OptiMem®. The cells were imaged at 37 °C (OKO lab incubation chamber) with a 3i Marianas spinning-disk confocal microscope equipped with a Zeiss AxioObserver Z1, a 40×/NA1.3 oil immersion objective, and a 3i Laserstack as excitation light source (568 nm). Emitted light was recorded through a band bass filter (Yokogawa CSU-X filter wheel) by a CMOS at 1152 × 1656 pixel size. Time-lapse images were recorded at 2-s intervals for 3 min (acquisition time, 500 ms). Elliptical regions of interest were stacked, and fluorescence intensities were analyzed using ImageJ program. The data obtained from 13–30 cells was plotted and analyzed in OriginPro 9. Fluorescence decay times were determined from single exponential fits to the data.

### Subcloning of RGECI into hippocampal expression vector

The RGECI variants were each subcloned into an expression vector (pCI) under the control of the human synapsin1 promoter by PCR using the following primers (NcoI-pCI-RCaMP-fw, EcoRI-pCI-RGECO-fw, and NotI-rv) and restriction ligation using NcoI/NotI for jRCaMP1a variants and EcoRI/NotI for jRGECO1a variants and T4 DNA ligase. Successful cloning was confirmed by DNA sequencing (Genewiz®): NcoI-pCI-RCaMP-fw, TCCACCATGGTGCAGAACGAGCTTGCTCTTAAG; and EcoRI-pCI-RGECO-fw, CATGCAGAATTCATGCTGCAGAACGAGCTTGCTCTTAAG.

### Single-cell electroporation

Organotypic hippocampal slices were prepared from Wistar rats at postnatal day 5 as described previously (34). The red GECIs plasmids were diluted to 100 ng/μl (pCI-syn-jRGECO1a, pCI-syn-jRCaMP1a, and their respective variants) and pCI-syn-mEmeraldGFP (a cytoplasmic GFP) was diluted to 10 ng/μl in potassium gluconate–based solution consisting of 135 mm potassium gluconate, 4 mm MgCl_2_, 4 mm Na_2_-ATP, 0.4 mm Na-GTP, 10 mm Na_2_-phosphocreatine, 3 mm ascorbate, and 10 mm HEPES, pH 7.2. CA3 pyramidal neurons were co-transfected by single cell electroporation at days 10–15 in culture with a mixture of the RGECI and the green morphological marker. For the electroporation procedure, slice cultures were kept in 145 mm NaCl, 25 mm d-glucose, 2.5 mm KCl, 1 mm MgCl_2_, 2 mm CaCl_2_, and 10 mm HEPES, pH 7.4 (sterile filtered). Thin-wall glass pipettes with a resistance of 12–14 MΩ were filled with a potassium gluconate–based solution containing the mixture of the two plasmids. An Axoporator 800A (Molecular Devices) was used to deliver 50 voltage pulses (−12 V, 0.5 ms) at 50 Hz (35).

### Solution and electrophysiology

The experiments were performed 8–10 days after electroporation when 50 ng/μl of red GECI was electroporated or 3–5 days after electroporation when 100 ng/μl of red GECI was electroporated. Recordings were performed in a recording solution containing 135 mm NaCl, 12.5 mm d-glucose, 1 mm NaH_2_PO_4_, 2.5 mm KCl, 4 mm MgCl_2_, 4 mm CaCl_2_, and 10 mm Na^+^-HEPES, pH 7.4, at 24 °C. The synaptic blockers d-APV (50 μm), NBQX (10 μm), and picrotoxin (100 μm) were added to the recording solution. Patch pipettes with a tip resistance of 3.5–4.5 mΩ were filled with 135 mm potassium gluconate, 4 mm MgCl_2_, 4 mm Na_2_-ATP, 0.4 mm Na-GTP, 10 mm Na_2_-phospho-creatine, 3 mm ascorbate, and 10 mm HEPES, pH 7.2. Whole-cell recordings from transfected CA3 pyramidal neurons were made with a Multiclamp 700B amplifier (Molecular Devices) under the control of Ephus software written in Matlab (36). The analog signals were filtered at 6 kHz and digitized at 10 kHz. CA3 neurons were held in current clamp and stimulated through the patch pipette by brief electrical pulses (2 ms, 3.0 nA) to induce a burst of 10 action potentials at 100 Hz. Trials were repeated at a frequency of 0.1 Hz.

### Two-photon microscopy

The custom-built two-photon imaging setup was based on an Olympus BX51WI microscope controlled by a customized version of the open-source software package ScanImage (37) written in MATLAB (MathWorks). A pulsed Ti:Sapphire laser (MaiTai DeepSee, Spectra Physics) tuned to 1040 nm was used to excite the RGECI. Red fluorescence was detected through the objective (LUMPLFLN 60XW, 60×, 1.0 NA, Olympus) and through the oil immersion condenser (1.4 NA, Olympus) by photomultiplier tubes (H7422P-40SEL, Hamamatsu). 560 DXCR dichroic mirrors and 607/70 emission filters (Chroma Technology) were used to separate red fluorescence. Excitation light was blocked by short-pass filters (ET700SP-2P, Chroma). ScanImage was modified for the user to freely define the circle scan across individual spines. Photomultiplier dark noise was measured before shutter opening and subtracted for every trial. jRGECO1a and variants displayed strong photoswitching during imaging at 1040 nm (increase in brightness). Therefore, imaging was started 2 s before stimulation to maximize the brightness of jRGECO1a and variants. To correct for bleaching, a monoexponential or double exponential decay was fitted to F0 in nonstimulated trials (deinterleaved with the 10 backpropagating action potentials) and then subtracted to the signal of individual trials.

## Author contributions

S. K. and C. C. data curation; S. K., C. C., and C. D. D. formal analysis; S. K. and C. D. D. investigation; S. K. and K. T. methodology; S. K. and K. T. writing-original draft; S. K. and K. T. writing-review and editing; T. G. O. and K. T. conceptualization; T. G. O. and K. T. supervision; T. G. O. validation; K. T. resources; K. T. funding acquisition.

## Supplementary Material

Supporting Information

## Footnotes

2 The abbreviations used are:

GECI: genetically encoded calcium indicator
RGECI: red-fluorescent GECI
cp: circularly permuted
FP: fluorescent protein
RFP: red fluorescent proteins
CaM: calmodulin
BAPTA: 1,2-bis(*o*-aminophenoxy)-ethane-*N*,*N*,*N*′,*N*′-tetraacetic acid
bAP: backpropagating action potential.
