# Supplementary material for: The kinetic mechanisms of fast-decay red-fluorescent genetically encoded calcium indicators
Source: J Biol Chem. 2019 Jan 16;294(11):3934–46. doi: 10.1074/jbc.RA118.004543 (PMC6422079; doi:10.1074/jbc.RA118.004543)
Supplement: Supporting Information [file supp_294_11_3934__index.html]

The kinetic mechanisms of fast-decay red-fluorescent genetically-encoded calcium indicators — Fast-RGECI kinetics — The kinetic mechanisms of fast-decay red-fluorescent genetically encoded calcium indicators — Fast RGECI kinetics — Supporting Information 

# The kinetic mechanisms of fast-decay red-fluorescent genetically encoded calcium indicators

## Supporting Information

- Supporting Information (to be published online) - Supporting Information to be published online
