# Supplementary material for: The kinetic mechanisms of fast-decay red-fluorescent genetically encoded calcium indicators
Source: J Biol Chem. 2019 Jan 16;294(11):3934–46. doi: 10.1074/jbc.RA118.004543 (PMC6422079; doi:10.1074/jbc.RA118.004543)
Supplement: Supporting Information [file supp_RA118.004543_138936_3_supp_267497_pl8888.docx]

The kinetic mechanisms of fast-decay red-fluorescent genetically-encoded calcium indicators

**Silke Kerruth^1^, Catherine Coates^1^, Céline D. Dürst^2^, Thomas G. Oertner^2^ and Katalin Török^1*^**

From the ^1^Molecular and Clinical Sciences Research Institute, St George’s, University of London, Cranmer Terrace, London SW17 0RE, United Kingdom and ^2^Institute for Synaptic Physiology, Center for Molecular Neurobiology Hamburg, 20251 Hamburg, Germany

*To whom correspondence should be addressed: Katalin Török: ^1^Molecular and Clinical Sciences Research Institute, St George’s, University of London, Cranmer Terrace, London SW17 0RE, United Kingdom; [k.torok@sgul.ac.uk](mailto:k.torok@sgul.ac.uk); Tel. (0044) 0208 725 5832; Fax. (0044) 020 8725 3581

**Supporting Information (SI)**

**List of materials included**

*Sequence alignment of cpmRuby from jRCaMP1a and mApple from jRGECO1a.*

The middle row show the residues that are conserved with their one-letter code and residues that have similar side groups with a ‘+’. The linker is marked in green and the fluorophore forming residues are highlighted in red.

Ruby: 3 EMMYPADGGLRGYTHMALKVDGGGHLSCSFVTTYRSKKTVGNIKMPAIHSVSHRLERLEE 62

E MYP DG L+ L++ GGH + TTY++KK V ++P + V +L+ +

Apple: 3 ERMYPEDGALKSEIKKGLRLKDGGHYAAEVKTTYKAKKPV---QLPGAYIVDIKLDIVSH 59

Ruby: 63 SDNEMFVVQREHAVAKFVGLGG-----GGGTGGSMNS--------LIKENMRMKVVLEGS 109

+++ V Q E A + GG GGTGGS+ S +IKE MR KV +EGS

Apple: 60 NEDYTIVEQCERAEGRH-STGGMDELYKGGTGGSLVSKGEEDNMAIIKEFMRFKVHMEGS 118

Ruby: 110 VNGHQFKCTGEGEGNPYMGTQTMRIKVIEGGPLPFAFDILATSFMYGSRTFIKYPKGIPD 169

VNGH+F+ GEGEG PY QT ++KV +GGPLPFA+DIL+ FMYGS+ +IK+P IPD

Apple: 119 VNGHEFEIEGEGEGRPYEAFQTAKLKVTKGGPLPFAWDILSPQFMYGSKAYIKHPADIPD 178

Ruby: 170 FFKQSFPEGFTWERVTRYEDGGVITVMQDTSLEDGCLVYHVQVRGVNFPSNGAVMQKKTK 229

+FK SFPEGF WERV +EDGG+I V QD+SL+DG +Y V++RG NFP +G VMQKKT

Apple: 179 YFKLSFPEGFRWERVMNFEDGGIIHVNQDSSLQDGVFIYKVKLRGTNFPPDGPVMQKKTM 238

Ruby: 230 GWE 232

GWE

Apple: 239 GWE 241


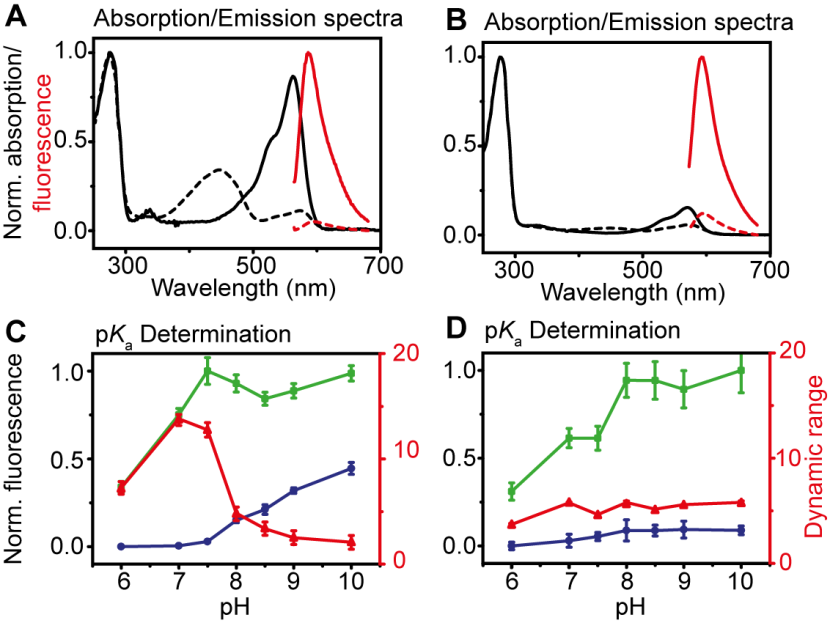


**SI Figure S1.** Absorption () and fluorescence emission spectra () of (A) jRGECO1a and (B) jRCaMP1a in the presence (solid lines) and absence of Ca^2+^ (dashed lines). Absorption spectra were normalized to 1 for absorption at 280 nm. Fluorescence emission spectra were normalized to the maximum emission with Ca^2+^. The absorption at 280 nm of the aromatic amino acids as well as the absorption at 570 nm of the intrinsic chromophore are visible. In the absorption spectra of jRGECO1a and its variants measured in assay buffer containing 2 mM EGTA (dashed line) a third band at 450 nm is visible. pH sensitivity and p*K*_a_ determination for (C) jRGECO1a and (D) jRCaMP1a, respectively. Shown are the normalized maxima of the fluorescence emission spectra at a range of pH values measured in assay buffer containing 1 mM CaCl_2_ () or 2 mM BAPTA (). The dynamic ranges were calculated using these maxima of the emission spectra in the presence (*F*_+Ca_^2+^) and absence of Ca^2+^ (*F*_-Ca_^2+^) (). The error bars represent standard deviation (SD).


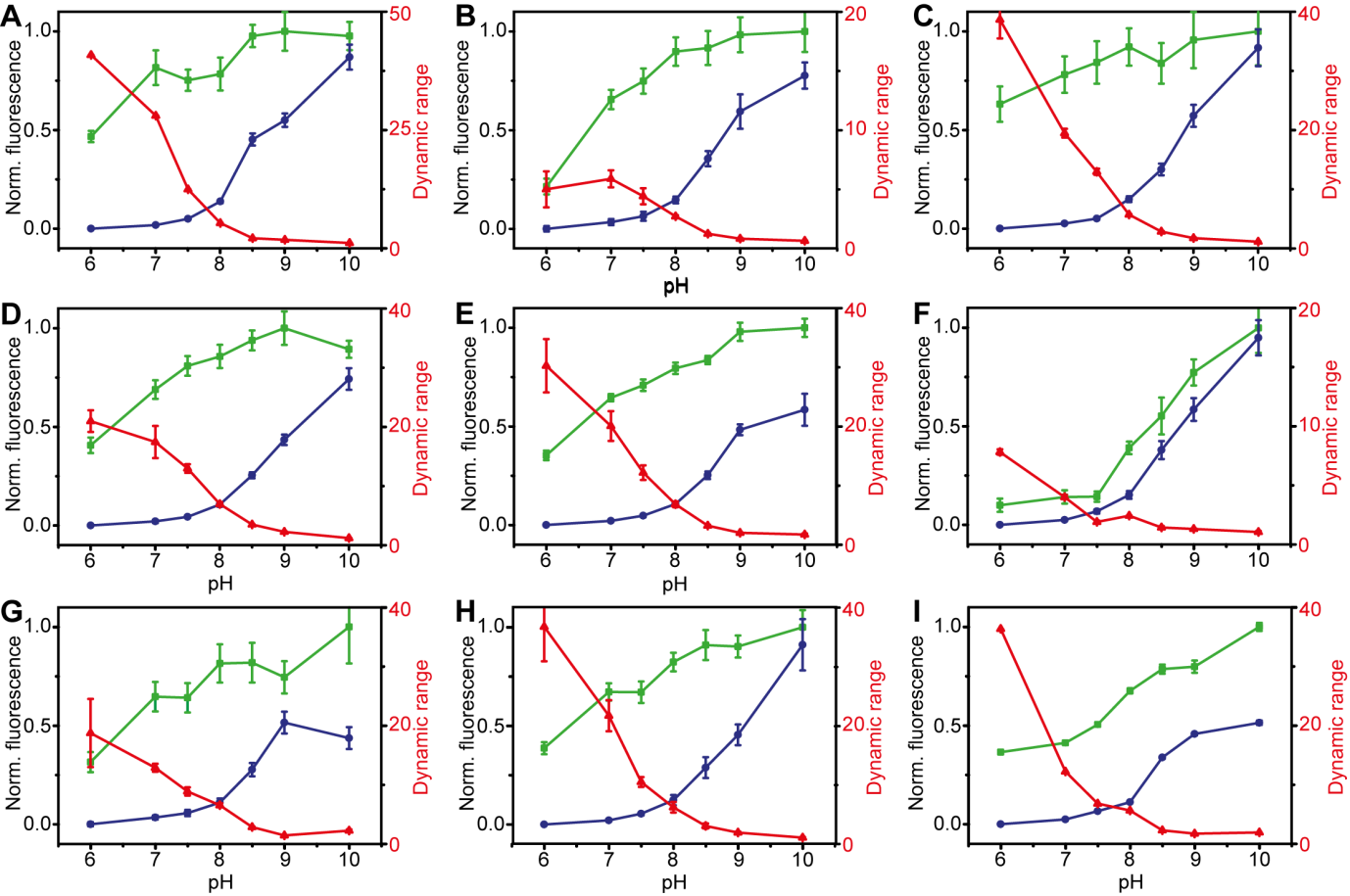


**SI Figure S2.** p*K*a determination of (A) jRGECO1a RS-1, (B) f-RGECO1, (C) jRGECO1a EF-2, (D) jRGECO1a EF-3, (E) jRGECO1a EF-4, (F) jRGECO1a RS-1 EF-1, (G) jRGECO1a RS-1 EF‑2, (H) jRGECO1a RS-1 EF-3 and (I) f-RGECO2. Shown are the normalized fluorescence maxima at a range of pH values measured in assay buffer containing 1 mM CaCl_2_ () and in 2 mM BAPTA (). The calculated dynamic range is shown in red (). The error bars represent standard deviation (SD).


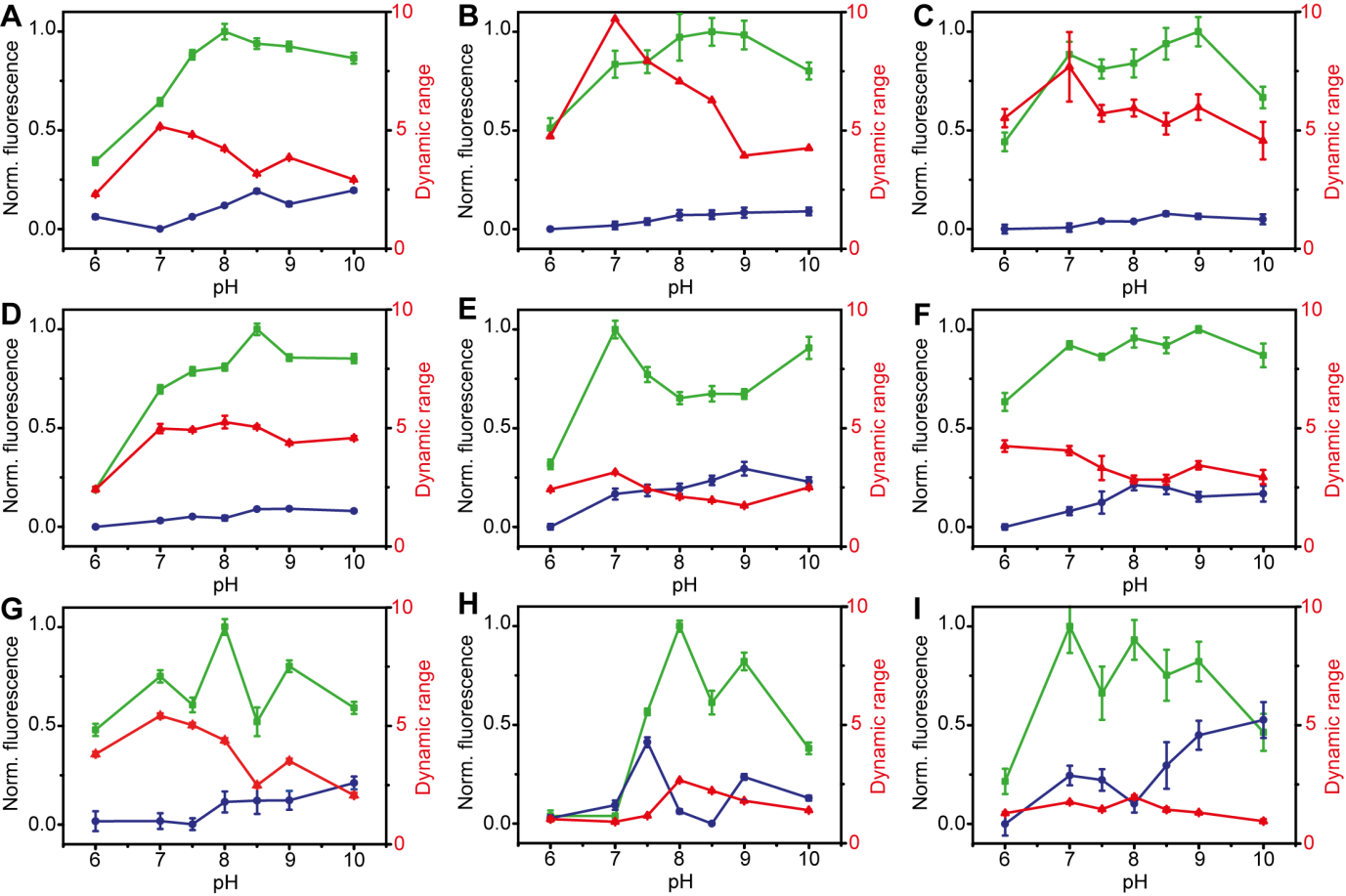


**SI Figure S3.** p*K*a determination of (A) f-RCaMP1, (B) jRCaMP1a EF-1, (C) jRCaMP1a EF-2, (D) f-RCaMP2, (E) jRCaMP1a EF-4, (F) jRCaMP1a RS-1 EF-1, (G) jRCaMP1a RS-1 EF‑2, (H) jRCaMP1a RS-1 EF-3 and (I) jRCaMP1a RS-1 EF-4. Shown are the normalized fluorescence maxima at a range of pH values measured in assay buffer containing 1 mM CaCl_2_ () and in 2 mM BAPTA (). The calculated dynamic range is shown in red (). The error bars represent standard deviation (SD).


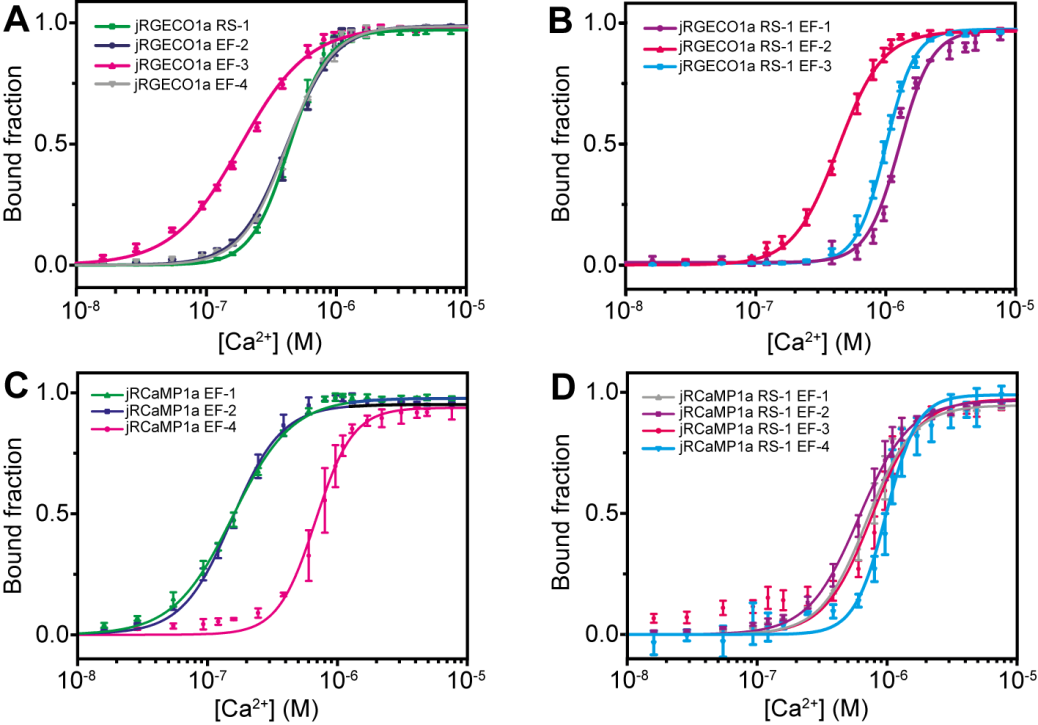


**SI Figure S4.** Equilibrium Ca^2+^ titration of jRGECO1a and jRCaMP1a variants. The data were fitted with a Hill slope for one specific site (lines). (A) jRGECO1a RS-1 (), jRGECO1a EF‑2 (), jRGECO1a EF-3 () and jRGECO1a EF-4 (). (B) jRGECO1a RS-1 EF-1 ( ), jRGECO1a RS-1 EF-2 () and jRGECO1a RS-1 EF-3 (). (C) jRCaMP1a EF-1 ( ), jRCaMP1a EF-2 () and jRCaMP1a EF-4 (). (D) jRCaMP1a RS-1 EF-1 (), jRCaMP1a RS-1 EF-2 (), jRCaMP1a RS-1 EF-3 () and jRCaMP1a RS-1 EF-4 (). The error bars represent standard deviation (SD).


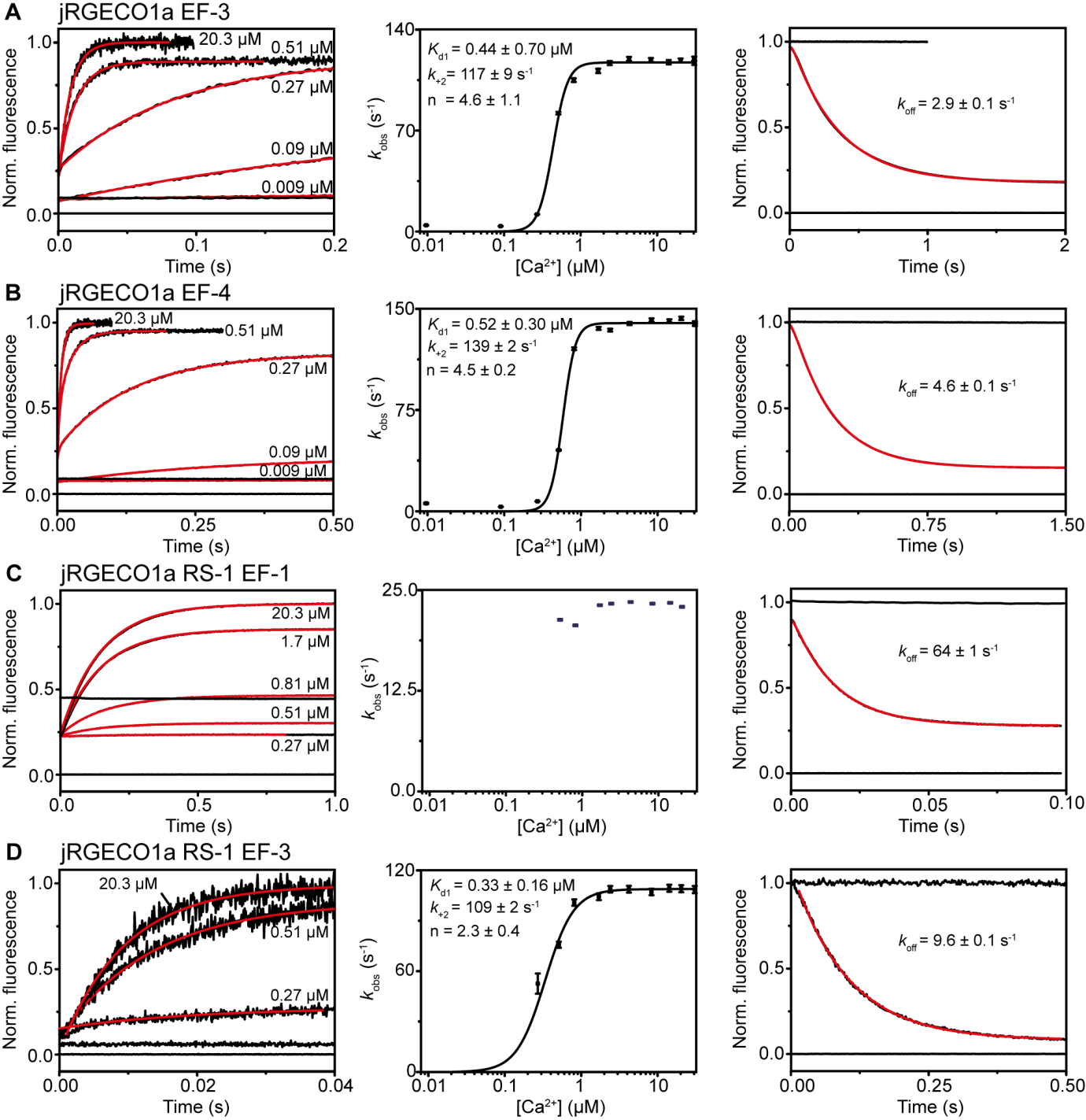


**SI Figure S5.** Kinetics of jRGECO1a variants that follow the kinetic model 1 like jRGECO1a: (A) jRGECO1a EF-3, (B) jRGECO1a EF-4, (C) jRGECO1a RS-1 EF-1 and (D) jRGECO1a RS-1 EF‑3. On the left panels the time traces for the association (black lines) at different [Ca^2+^] (final concentration is given for each trace) with the fitted curves (red lines) are shown. The straight black line at zero represents the time trace when buffer is mixed with buffer while the other straight line with the offset is the time trace when the RGECI is mixed with buffer containing 10 mM EGTA. In the middle panel the observed time constants *k*_obs_ of the association traces are plotted against the [Ca^2+^]. The data were fitted to **Scheme 1**. The parameters obtained with the fit are displayed inside the plot. On the right panel the dissociation time traces are shown (black lines) with their corresponding fits (red lines). The observed dissociation rates are displayed next to the graphs. The zero lines represent the time traces when buffer is mixed with buffer while the straight lines at 1 are the time traces when the RGECI are mixed with assay buffer containing 1 mM CaCl_2_.


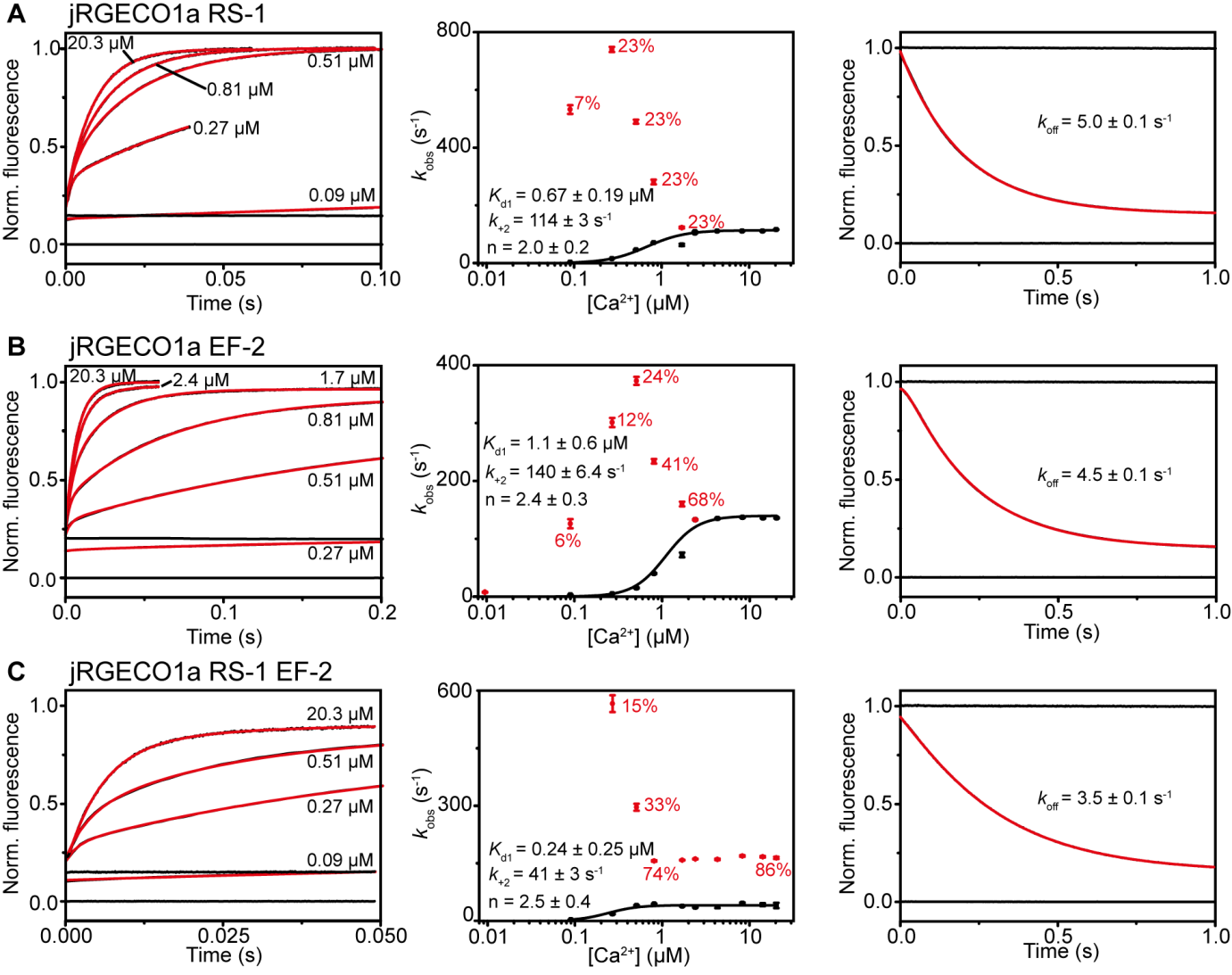


**SI Figure S6.** Kinetics of jRGECO1a variants that follow kinetic model 2: (A) jRGECO1a RS-1, (B) jRGECO1a EF‑2 and (C) jRGECO1a RS-1 EF-2. On the left panel the time traces for the association (black lines) at different free [Ca^2+^] (final concentration is given for each trace) with the fitted curves (red lines) are shown. The straight black line at zero represents the time trace when buffer is mixed with buffer while the other straight line with the offset is the time trace when the RGECI is mixed with buffer containing 10 mM EGTA. In the middle panel the observed time constants *k*_obs_ of the association traces are plotted against the [Ca^2+^]. The slow rates were fitted to **Scheme 1**. The fast phase show similar behaviour like jRGECO1a EF-2. The parameters obtained with the fit are displayed inside the panel. On the right panel the dissociation time traces are shown (black lines) with their corresponding fits (red lines). The observed dissociation rates are displayed next to the graphs. The zero lines represent the time traces when buffer is mixed with buffer while the straight lines at 1 are the time traces when the RGECI is mixed with assay buffer containing 1 mM CaCl_2_.


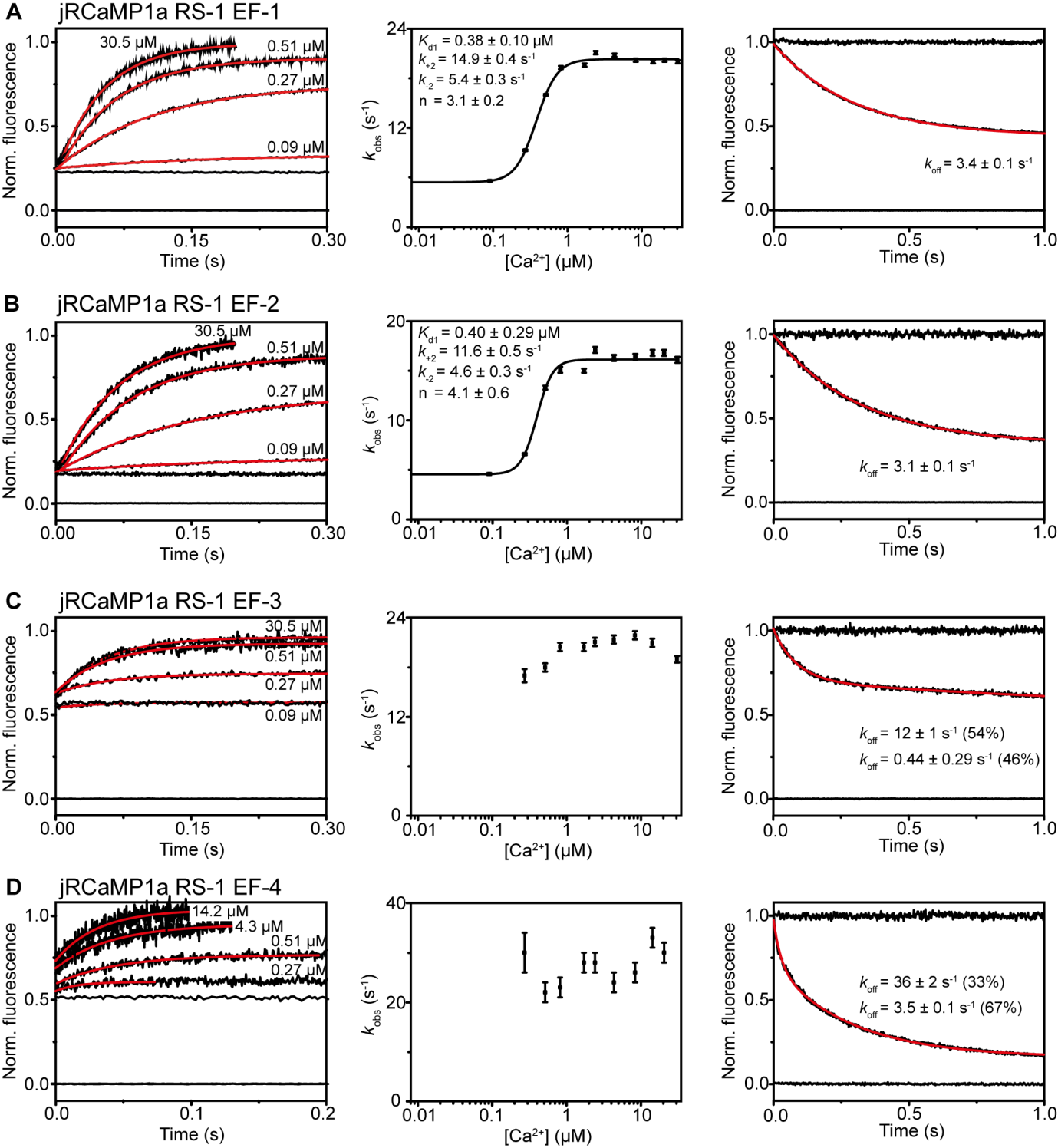


**SI Figure S7.** Kinetics of jRCaMP1a variants following **Scheme 2**. (A) jRCaMP1a RS-1 EF‑1, (B) jRCaMP1a RS-1 EF-2, (C) jRCaMP1a RS-1 EF-3 and (D) jRCaMP1a RS-1 EF-4. On the left panel the time traces for the association (black lines) at different [Ca^2+^] (final concentration is given for each trace) with the fitted curves (red lines) are shown. The straight black line at zero represents the time trace when buffer is mixed with buffer while the other straight line with the offset is the time trace when the RGECI is mixed with buffer containing 10 mM EGTA. In the middle panel the observed time constants *k*_obs_ of the association traces are plotted against the [Ca^2+^]. When possible the data was fitted to **Scheme 1 with reversible second stage**. The parameters obtained with the fit are displayed inside the panel. On the right panel the dissociation time traces are shown (black lines) with their corresponding fits (red lines). The observed dissociation rates are states displayed next to the graphs with their relative amplitudes shown in brackets. The zero lines represent the time traces when buffer is mixed with buffer while the straight lines at 1 are the time traces when the RGECI is mixed with buffer containing 1 mM CaCl_2_.


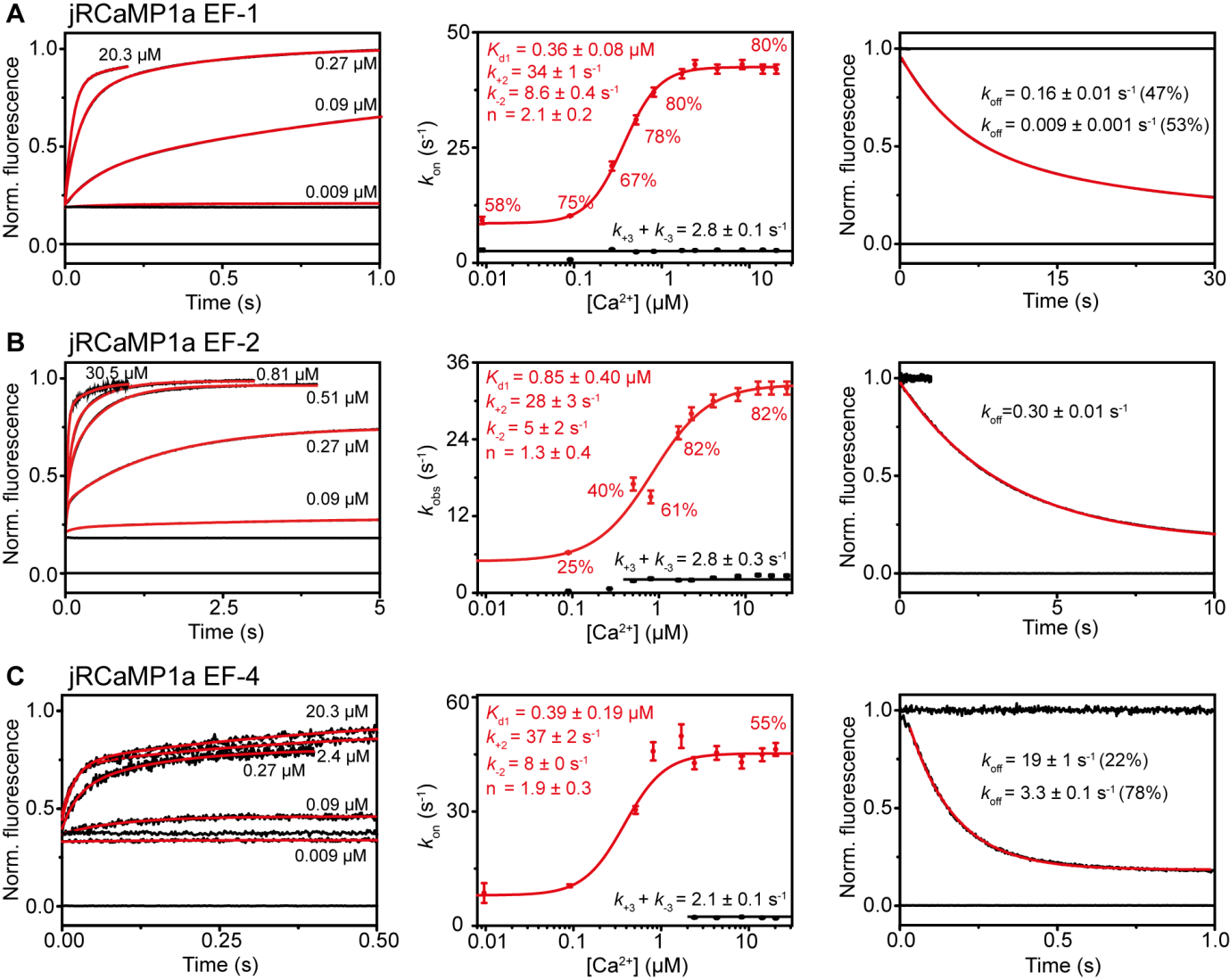


**SI Figure S8.** Kinetics of jRCaMP1a variants (A) jRCaMP1a EF‑2, (B) jRCaMP1a EF-3 and (C) jRCaMP1a EF-4. On the left panel the time traces for the association (black lines) at different [Ca^2+^] (final concentration is given for each trace) with the fitted curves (red lines) are shown. The straight black line at zero represents the time trace when buffer is mixed with buffer while the other straight line with the offset is the time trace when the RGECI is mixed with buffer containing 10 mM EGTA. In the middle panel the observed time constants *k*_obs_ of the association traces are plotted against the [Ca^2+^]. The fast rates are shown in red and were fitted with to **Scheme 1 with back reaction** with the obtained parameters shown in red next to the plot. The slow rates are shown in black and the limiting rates are shown. On the right panel the dissociation time traces are shown (black lines) with their corresponding fits (red lines). The observed dissociation rates are states displayed next to the graphs with their relative amplitudes shown in brackets. The zero lines represent the time traces when buffer is mixed with buffer while the straight lines at 1 are the time traces when the RGECI is mixed with buffer containing 1 mM CaCl_2_.


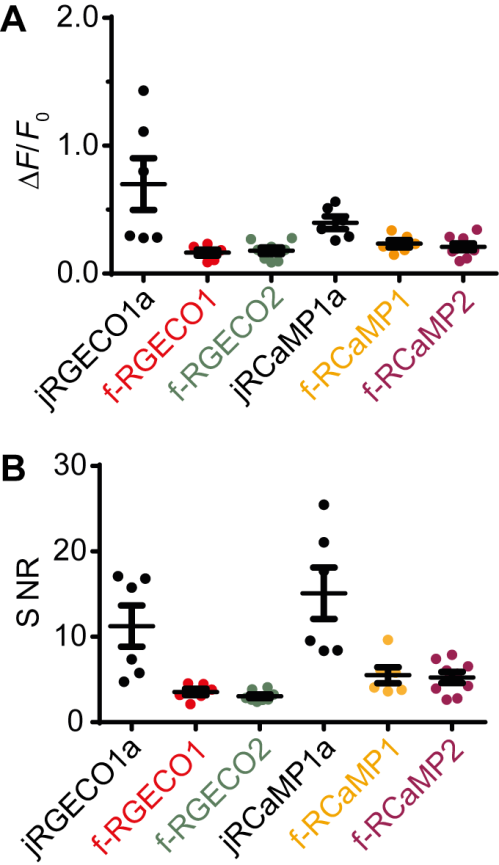


**SI Figure S9**. Peak amplitude and signal-to-noise ratio (SNR) of Ca^2+^ transients observed in hippocampal slices using RGECIs. (A) Peak amplitudes of individual spines in response to 10 backpropagating action potentials (bAP) of CA3 neurons expressing jRGECO1a (Δ*F*/*F*_0_ = 0.7 ± 0.2; n = 6 spines), f-RGECO1 (Δ*F*/*F*_0_ = 0.16 ± 0.02; n = 6 spines), f-RGECO2 (Δ*F*/*F*_0_ = 0.18 ± 0.03; n = 8 spines); jRCaMP1a (Δ*F*/*F*_0_ = 0.40 ± 0.05; n = 6 spines); f‑RCaMP1 (Δ*F*/*F*_0_ = 0.23 ± 0.03; n = 6 spines); f-RCaMP2 (Δ*F*/*F*_0_ = 0.21 ± 0.03; n = 9 spines). (B) Signal-to-noise ratio (SNR = mean peak amplitude / STDEV (*F*_0_)) of individual spines in response to 10 bAP of CA3 neurons expressing jRGECO1a (SNR = 11 ± 3; n = 6 spines), f-RGECO1 (SNR = 3.5 ± 0.4; n = 6 spines), f-RGECO2 (SNR = 3.0 ± 0.3; n = 8 spines); jRCaMP1a (SNR = 15 ± 4; n = 6 spines); f‑RCaMP1 (SNR = 5.5 ± 1.0; n = 6 spines); f-RCaMP2 (SNR = 5.2 ± 0.7; n = 9 spines). Values are expressed as mean ± s.e.m.

**SI Table S1A.**

| **variant** | **dynamic range *F*_+Ca_2+/*F*_-Ca_2+** | ***K*_d_ (nM)** | ***n*** | ***k*_on(lim)_ (s^-1^)** | ***k*_off_ (s^‑1^)** |
| --- | --- | --- | --- | --- | --- |
| jRGECO1a | 13.8 ± 2.9 | 162 ± 2 | 1.8 ± 0.1 | 150 ± 3 | 3.4 ± 0.1 |
|  |  |  |  |  |  |
| jRGECO1a RS-1 | 14.1 ± 1.7 | 428 ± 4 | 3.2 ± 0.1 | 111 ± 1 | 5.0 ± 0.1 |
|  |  |  |  |  |  |
| jRGECO1a EF-2 | 15.4 ± 3.6 | 436 ± 5 | 2.6 ± 0.1 | 136 ± 1 | 4.5 ± 0.1 |
|  |  |  |  |  |  |
| jRGECO1a EF-3 | 10.9 ± 2.0 | 187 ± 3 | 1.7 ± 0.1 | 118 ± 1 | 2.9 ± 0.1 |
|  |  |  |  |  |  |
| jRGECO1a EF-4 | 12.7 ± 1.7 | 425 ± 4 | 2.7 ± 0.1 | 140 ± 2 | 4.6 ± 0.1 |
|  |  |  |  |  |  |
| jRGECO1a RS-1 EF-1 | 6.2 ± 1 | 1216 ± 21 | 3.9 ± 0.3 | 23 ± 1 | 64 ± 1 |
|  |  |  |  |  |  |
| jRGECO1a RS-1 EF-2 | 6.3 ± 2.6 | 429 ± 5 | 2.5 ± 0.1 | 42 ± 2 (15%)  173 ± 2 (85%) | 3.5 ± 0.1 |
|  |  |  |  |  |  |
| jRGECO1a RS-1 EF-3 | 16.8 ± 6.2 | 973 ± 7 | 3.5 ± 0.1 | 109 ± 2 | 9.6 ± 0.1 |
|  |  |  |  |  |  |

**SI table S1B.**

| **variant** | **p*K*_a_** | | **Φ** | | **ε_570nm_ (mM^‑1^cm^‑1^)** | | **Brightness (mM^‑1^cm^‑1^)** | |
| --- | --- | --- | --- | --- | --- | --- | --- | --- |
|  | + Ca^2+^ | - Ca^2+^ | + Ca^2+^ | - Ca^2+^ | + Ca^2+^ | - Ca^2+^ | + Ca^2+^ | - Ca^2+^ |
| jRGECO1a | 6.4 ± 0.1 | 8.5 ± 0.1 | 0.220 | 0.120 | 41.0 ± 5.6 | 5.6 ± 0.8 | 9.0 ± 0.7 | 0.7 ± 0.3 |
| jRGECO1a RS-1 | 6.2 ± 0.1 | 8.7 ± 0.2 | 0.222 ± 0.015 | 0.111 ± 0.032 | 26.1 ± 0.6 | 4.2 ± 0.7 | 5.8 ± 0.5 | 0.5 ± 0.3 |
| jRGECO1a EF-2 | 5.0 ± 0.5 | 8.9 ± 0.2 | 0.177 ± 0.009 | 0.099 ± 0.021 | 27.8 ± 4.2 | 2.6 ± 0.1 | 4.9 ± 1.0 | 0.3 ± 0.1 |
| jRGECO1a EF-3 | 6.3 ± 0.1 | 9.2 ± 0.1 | 0.187 ± 0.007 | 0.0145 ± 0.031 | 37.6 ± 4.0 | 4.5 ± 0.3 | 7.0 ± 1.0 | 0.7 ± 0.2 |
| jRGECO1a EF-4 | 6.5 ± 0.1 | 8.5 ± 0.1 | 0.239 ± 0.012 | 0.0167 ± 0.044 | 31.9 ± 1.0 | 4.0 ± 0.2 | 7.6 ± 0.7 | 0.7 ± 0.3 |
| jRGECO1a RS-1 EF-1 | 8.3 ± 0.2 | 8.8 ± 0.2 | 0.261 ± 0.015 | 0.160 ± 0.028 | 14.0 ± 0.3 | 3.5 ± 0.1 | 3.7 ± 0.3 | 0.6 ± 0.1 |
| jRGECO1a RS-1 EF-2 | 6.6 ± 0.1 | 8.5 ± 0.3 | 0.202 ± 0.004 | 0.160 ± 0.028 | 14.4 ± 5.6 | 2.8 ± 0.1 | 2.9 ± 1.2 | 0.5 ± 0.1 |
| jRGECO1a RS-1 EF-3 | 6.4 ± 0.1 | 9.1 ± 0.1 | 0.289 ± 0.027 | 0.152 ± 0.046 | 31.9 ± 2.4 | 3.5 ± 0.6 | 9.2 ± 1.6 | 0.5 ± 0.3 |

**SI Table 2A.**

| **variant** | **dynamic range *F*_+Ca_2+/*F*_-Ca_2+** | ***K*_d_ (nM)** | ***n*** | ***k*_on(lim)_ (s^-1^)** | ***k*_off_ (s^‑1^)** |
| --- | --- | --- | --- | --- | --- |
| jRCaMP1a | 7.0 ± 2.1 | 141 ± 3 | 1.5 ± 0.1 | 56 ± 1 (47%)  2.2 ± 0.1 (53%) | 2.2 ± 0.1 (17%)  0.32 ± 0.01 (83%) |
|  |  |  |  |  |  |
| jRCaMP1a EF-1 | 4.6 ± 1.2 | 156 ± 3 | 1.9 ± 0.1 | 42 ± 1 (80%)  2.7 ± 0.1 (20%) | 0.16 ± 0.01 (47%)  0.009 ± 0.001 (53%) |
|  |  |  |  |  |  |
| jRCaMP1a EF-2 | 5.6 ± 1.8 | 154 ± 3 | 2.3 ± 0.1 | 32 ± 1 (83%)  2.8 ± 0.1 (17%) | 0.30 ± 0.01 |
|  |  |  |  |  |  |
| jRCaMP1a EF-4 | 2.2 ± 0.2 | 688 ± 15 | 3.0 ± 0.2 | 46 ± 2 (56%)  2.0 ± 0.1 (44%) | 19 ± 1 (22%)  3.3 ± 0.1 (78%) |
|  |  |  |  |  |  |
| jRCaMP1a RS-1 EF-1 | 3.7 ± 1.1 | 702 ± 12 | 2.4 ± 0.1 | 20 ± 1 | 3.4 ± 0.1 |
|  |  |  |  |  |  |
| jRCaMP1a RS-1 EF-2 | 4.5 ± 1.3 | 607 ± 11 | 2.2 ± 0.1 | 16.8 ± 0.3 | 3.1 ± 0.1 |
|  |  |  |  |  |  |
| jRCaMP1a RS-1 EF-3 | 1.4 ± 0.4 | 768 ± 29 | 2.2 ± 0.1 | 21 ± 1 | 12 ± 1 (54%)  0.44 ± 0.29 (46%) |
|  |  |  |  |  |  |
| jRCaMP1a RS-1 EF-4 | 1.5 ± 0.1 | 980 ± 21 | 3.6 ± 0.3 | 30 ± 2 | 36 ± 2 (33%)  3.5 ± 0.1 (67%) |
|  |  |  |  |  |  |

**SI table S2B.**

| **variant** | **p*K*_a_** | | **Φ** | | **ε_570nm_ (mM^‑1^cm^‑1^)** | | **Brightness (mM^‑1^cm^‑1^)** | |
| --- | --- | --- | --- | --- | --- | --- | --- | --- |
|  | + Ca^2+^ | - Ca^2+^ | + Ca^2+^ | - Ca^2+^ | + Ca^2+^ | - Ca^2+^ | + Ca^2+^ | - Ca^2+^ |
| jRCaMP1a | 6.6 ± 0.1 | 7.5 ± 0.1 | 0.515 | 0.308 | 5.3 ± 0.6 | 1.7 ± 0.1 | 2.7 ± 0.4 | 0.5 ± 0.1 |
| jRCaMP1a EF-1 | 5.8 ± 0.3 | 7.9 ± 0.2 | 0.411 ± 0.150 | 0.188 ± 0.032 | 4.7 ± 0.3 | 2.4 ± 0.3 | 2.0 ± 0.9 | 0.4 ± 0.2 |
| jRCaMP1a EF-2 | 6.2 ± 0.1 | 8.0 ± 0.2 | 0.342 ± 0.090 | 0.189 ± 0.046 | 4.7 ± 0.1 | 2.3 ± 0.1 | 1.6 ± 0.5 | 0.4 ± 0.2 |
| jRCaMP1a EF-4 | 6.3 ± 0.1 | 8.0 ± 0.2 | 0.431 ± 0.114 | 0.331 ± 0.067 | 1.0 ± 0.1 | 0.7 ± 0.1 | 0.5 ± 0.2 | 0.2 ± 0.1 |
| jRCaMP1a RS-1 EF-1 | 5.0 ± 0.5 | 7.5 ± 0.2 | 0.324 ± 0.089 | 0.210 ± 0.035 | 2.3 ± 0.1 | 1.3 ± 0.1 | 0.8 ± 0.3 | 0.3 ± 0.1 |
| jRCaMP1a RS-1 EF-2 | 6.1 ± 0.1 | 7.9 ± 0.3 | 0.39 ± 0.105 | 0.235 ± 0.040 | 5.0 ± 0.2 | 2.8 ± 0.1 | 2.0 ± 0.6 | 0.7 ± 0.2 |
| jRCaMP1a RS-1 EF-3 | 7.4 ± 0.1 | 8.0 ± 0.5 | 0.381 ± 0.100 | 0.400 ± 0.066 | 2.5 ± 0.1 | 2.1 ± 0.2 | 0.9 ± 0.3 | 0.8 ± 0.2 |
| jRCaMP1a RS-1 EF-4 | 6.4 ± 0.1 | 8.3 ± 0.2 | 0.339 ± 0.084 | 0.384 ± 0.065 | 0.54 ± 0.05 | 0.36 ± 0.04 | 0.18 ± 0.06 | 0.14 ±0.04 |

**Scheme 1 irreversible isomerisation**

Based on a model the rate equations for the formation of the fluorescent complex can be formulated. For the formation of the fully bound fluorescent complex the following equation is formulated:


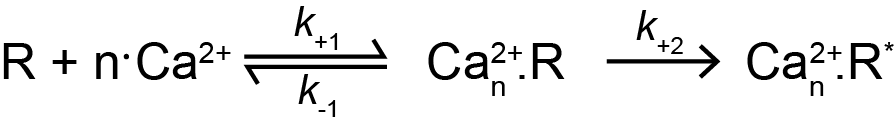


With R as the RGECI species, Ca_n_^2+^.R representing the fully Ca^2+^ bound complex and Ca_n_^2+^.R* representing the fully Ca^2+^ and RS-20 bound to CaM fluorescent complex.

We assume a pre-equilibrium state between the free reactants RGECI (R) and Ca^2+^ with the equilibrium constant *K*_1_:

$K_{1}=\frac{k_{+1}}{k_{-1}}=\frac{[{Ca}_{n}^{2+}.R]}{\left[ R \right][{Ca}^{2+}]^{n}}$ with the dissociation constant defined as $K_{d1}=\sqrt[n]{\frac{1}{K_{1}}}$ Eq. S1

The rate equation for the fully Ca^2+^ bound complex Ca_n_^2+^.R is shown in equation (S2).

$\frac{\partial[{Ca}_{n}^{2+}.R]}{\partial t}=k_{1}\left[ R \right][{Ca}^{2+}]^{n}-k_{-1}[{Ca}_{n}^{2+}.R]$ Eq. S2

Assuming a quasi-stationary state for the complex, equation (S2) is equal to zero and can be solved to the complex concentration, resulting in equation (S3)

$\left[ {Ca}_{n}^{2+}.R \right]=K_{1}[R][{Ca}^{2+}]^{n}$ Eq. S3

The overall concentration of RGECI [R]_0_ is the sum of all complexes (Ca_n_^2+^.R and Ca_n_^2+^.R*) and the unbound RGECI (R), as shown in equation (4):

$[R]_{0}=\left[ R \right]+\left[ {Ca}_{n}^{2+}.R \right]+[{Ca}_{n}^{2+}.R^{*}]$ Eq. S4

Inserting equation (S3) into equation (S4) and solving it towards [R] results in equation (S5).

$[R]=\frac{[R]_{0}-[{Ca}_{n}^{2+}.R^{*}]}{{1+K}_{1}[{Ca}^{2+}]^{n}}$ Eq. S5

Back inserting equation (S5) into equation (S3) gives an expression [Ca_n_^2+^.R] just in dependence of constants and of [Ca_n_^2+^.R*] (see equation (S6)).

$\left[ {Ca}_{n}^{2+}.R \right]=\frac{K_{1}[{Ca}^{2+}]^{n}[R]_{0}}{{1+K}_{1}[{Ca}^{2+}]^{n}}-\frac{K_{1}[{Ca}^{2+}]^{n}[{Ca}_{n}^{2+}.R^{*}]}{{1+K}_{1}[{Ca}^{2+}]^{n}}$ Eq. S6

The reaction equation of the formation of the fluorescent complex Ca_n_^2+^.R* is shown in equation (S7).

$\frac{\partial[{Ca}_{n}^{2+}.R^{*}]}{\partial t}=k_{+2}\left[ {Ca}_{n}^{2+}.R \right]- k_{-2}[{Ca}_{n}^{2+}.R^{*}]$ Eq. S7

Inserting equation (S6) into (S7) and performing the partial derivation leads to the final fitting equation (S8) for the slow observed rates in dependence on [Ca^2+^].

$k_{obs}=\frac{k_{+2}K_{1}[{Ca}^{2+}]^{n}}{{1+K}_{1}[{Ca}^{2+}]^{n}}$ Eq. S8

Further the amplitude A, the overall rate constant *K*_0_ and the overall *K*_d_ can be defined (see equations (S9a, b, c).

$A=\frac{k_{+2}K_{1}[R]_{0}[{Ca}^{2+}]^{n}}{{1+K}_{1}[{Ca}^{2+}]^{n}}$ Eq. S9a

$K_{0}=\frac{k_{+1}+k_{+2}}{k_{-1}}$ Eq. S9b

$K_{d}=\frac{k_{-1}}{k_{+1}+k_{+2}}$ Eq. S9c

**Scheme 1 reversible isomerisation**

If we assume that the formation of the fluorescent species is reversible kinetic model 1 changes to the following reaction equation:


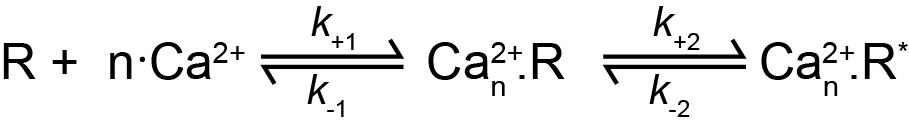


With the final fitting equation:

$k_{obs}=\frac{k_{+2}K_{1}[{Ca}^{2+}]^{n}}{{1+K}_{1}[{Ca}^{2+}]^{n}}+k_{-2}$ Eq. S10

Further the amplitude A, the overall rate constant *K*_0_ and with it the overall *K*_d_ can be defined (see equations (9a, b, c).

$A=\frac{k_{+2}K_{1}[R]_{0}[{Ca}^{2+}]^{n}}{{1+K}_{1}[{Ca}^{2+}]^{n}}$ Eq. S11a

$K_{0}=K_{1}+K_{1}K_{2}$ Eq. S11b

$K_{d}=\frac{1}{K_{0}}=\frac{1}{K_{1}+K_{1}K_{2}}$ Eq. S11c

**SI Table 3A**

| **variant** | ***K*_d_ (nM)** | **n** | ***K*_d1_ (nM)** | **n** | ***k***_+2_ **(s^‑1^)** |
| --- | --- | --- | --- | --- | --- |
|  |  |  | **from fit with Eq. 1** | | |
| jRGECO1a | 162 ± 2 | 1.8 ± 0.1 | 345 ± 71 | 2.6 ± 0.1 | 150 ± 2 |
| jRGECO1a RS-1 | 428 ± 4 | 3.2 ± 0.1 |  |  |  |
| jRGECO1a EF-1 (f‑RGECO1) | 1213 ± 15 | 3.0 ± 0.2 | 340 ± 105 | 1.2 ± 0.4 | 14.5 ± 0.5 |
| jRGECO1a EF-2 | 436 ± 5 | 2.6 ± 0.1 |  |  |  |
| jRGECO1a EF-3 | 187 ± 3 | 1.7 ± 0.1 | 435 ± 699 | 4.6 ± 1.1 | 117 ± 9 |
| jRGECO1a EF-4 | 425 ± 4 | 2.7 ± 0.1 | 526 ± 222 | 4.5 ± 0.2 | 139 ± 2 |
| jRGECO1a RS-1 EF-1 | 1216 ± 21 | 3.9 ± 0.3 |  |  |  |
| jRGECO1a RS-1 EF-2 | 429 ± 5 | 2.5 ± 0.1 |  |  |  |
| jRGECO1a RS-1 EF-3 | 973 ± 7 | 3.5 ± 0.1 | 323 ± 156 | 2.3 ± 0.4 | 109 ± 2 |
| jRGECO1a RS-1 EF-4 (f-RGECO2) | 1261 ± 11 | 5.8 ± 0.3 | 1250 ± 313 | 2.7 ± 0.3 | 79 ± 3 |

**SI Table 3B**

| **variant** | ***K*_d_ (nM)** | **n** | ***K*_d1_ (nM)** | **n** | ***k***_+2_ **(s^‑1^)** | ***k***_-2_ **(s^‑1^)** | ***K*_d(overall)_ (nM)** |
| --- | --- | --- | --- | --- | --- | --- | --- |
|  |  |  | **from fit with Eq. 3** | | | | **from Eq. 4** |
| jRCaMP1a | 141 ± 3 | 1.5 ± 0.1 | 345 ± 131 | 2.0 ± 0.2 | 53 ± 2 | 5.3 ± 0.8 | 379 ± 156 |
| jRCaMP1a RS-1 (f‑RCaMP1) | 520 ± 12 | 2.3 ± 0.1 | 400 ± 144 | 3.5 ± 0.3 | 17 ± 1 | 5.1 ± 0.5 | 520 ± 196 |
| jRCaMP1a EF-1 | 156 ± 3 | 1.9 ± 0.1 | 357 ± 90 | 2.1 ± 0.2 | 34 ± 1 | 8.6 ± 0.4 | 447 ± 115 |
| jRCaMP1a EF-2 | 154 ± 3 | 2.3 ± 0.1 | 847 ± 403 | 1.3 ± 0.4 | 28 ± 3 | 5 ± 2 | 999 ± 630 |
| jRCaMP1a EF-3 (f‑RCaMP2) | 785 ± 12 | 3.5 ± 0.2 |  |  |  |  |  |
| jRCaMP1a EF-4 | 688 ± 15 | 3.0 ± 0.2 | 385 ± 193 | 1.9 ± 0.3 | 37 ± 2 | 8 ± 1 | 468 ± 243 |
| jRCaMP1a RS-1 EF-1 | 702 ± 12 | 2.4 ± 0.1 | 385 ± 104 | 3.1 ± 0.2 | 15 ± 1 | 5.4 ± 0.3 | 524 ± 145 |
| jRCaMP1a RS-1 EF-2 | 607 ± 11 | 2.2 ± 0.1 | 400 ± 304 | 4.1 ± 0.6 | 12 ± 1 | 4.6 ± 0.3 | 559 ± 427 |
| jRCaMP1a RS-1 EF-3 | 768 ± 29 | 2.2 ± 0.1 |  |  |  |  |  |
| jRCaMP1a RS-1 EF-4 | 980 ± 21 | 3.6 ± 0.3 |  |  |  |  |  |
